# Development of Berberine-Loaded Nanoparticles for Astrocytoma Cells Administration and Photodynamic Therapy Stimulation

**DOI:** 10.3390/pharmaceutics15041078

**Published:** 2023-03-27

**Authors:** Sergio Comincini, Federico Manai, Milena Sorrenti, Sara Perteghella, Camilla D’Amato, Dalila Miele, Laura Catenacci, Maria Cristina Bonferoni

**Affiliations:** 1 Department of Biology and Biotechnology, University of Pavia, 27100 Pavia, Italy; 2 Department of Drug Sciences, University of Pavia, 27100 Pavia, Italy

**Keywords:** PLGA-nanoparticles, glioblastoma, cancer, chitosan oleate, berberine, hydrophobic salts

## Abstract

Berberine (BBR) is known for its antitumor activity and photosensitizer properties in anti-cancer photodynamic therapy (PDT), and it has previously been favorably assayed against glioblastoma multiforme (GBM)-derived cells. In this work, two BBR hydrophobic salts, dodecyl sulfate (S) and laurate (L), have been encapsulated in PLGA-based nanoparticles (NPs), chitosan-coated by the addition of chitosan oleate in the preparation. NPs were also further functionalized with folic acid. All the BBR-loaded NPs were efficiently internalized into T98G GBM established cells, and internalization increased in the presence of folic acid. However, the highest mitochondrial co-localization percentages were obtained with BBR-S NPs without folic acid content. In the T98G cells, BBR-S NPs appeared to be the most efficient in inducing cytotoxicity events and were therefore selected to assess the effect of photodynamic stimulation (PDT). As a result, PDT potentiated the viability reduction for the BBR-S NPs at all the studied concentrations, and a roughly 50% reduction of viability was obtained. No significant cytotoxic effect on normal rat primary astrocytes was observed. In GBM cells, a significant increase in early and late apoptotic events was scored by BBR NPs, with a further increase following the PDT scheme. Furthermore, a significantly increased depolarization of mitochondria was highlighted following BBR-S NPs’ internalization and mostly after PDT stimulation, compared to untreated and PDT-only treated cells. In conclusion, these results highlighted the efficacy of the BBR-NPs-based strategy coupled with photoactivation approaches to induce favorable cytotoxic effects in GBM cells.

## 1. Introduction

Among malignant primary brain tumors in humans, glioblastoma multiforme (GBM, WHO grade IV) is the most common and aggressive condition [1]. Unfortunately, standardized therapeutic approaches, such as surgical tumor resection and hemo- and/or radiotherapy protocols, have not induced significant advances in patients’ prognoses [2,3]. Despite the introduction into clinics of more effective chemotherapeutic drugs, such as temozolomide or carmustine, their relatively complex pharmacokinetics, accomplished by the large extent of cytological heterogeneity and acquired resistance phenotypes, did not overcome the main oncological features of GBM [4,5,6]. Consequently, novel experimental strategies are urgently required to improve the survival of GBM patients [7].

Bioactive compounds isolated from plants, referred to as phytochemicals or nutraceuticals, are gaining increased attention to counteract some features of cancer cells. Among these compounds, aloe-emodin, berberine, camptothecin, curcumin, and quercetin, with their ability to regulate different molecular growth and progression pathways, have been investigated in different oncological contexts [8,9].

Importantly, these natural compounds are generally characterized by a relatively reduced level of cytotoxicity when administered to healthy cells [10,11,12]. One of these natural compounds, berberine (BBR), is extracted from *Rhizoma coptidis* herbs and was used for centuries in traditional Chinese medicine prescriptions [13,14]. Of note, it has been reported that BBR has exhibited antitumor activities in different cancers [15,16], including GBM [17,18]. Moreover, it has been demonstrated that BBR can be photoactivated and act as a photosensitizer in anti-cancer photodynamic therapy (PDT), in cervical cancer [19], in renal carcinoma cells [20], and in brain tumor cells [17,21].

However, due to BBR’s limited solubility, its reduced oral bioavailability [22], and P-glycoproteins’ exocytic activity at the cancer cell membranes [23], the overall therapeutic efficacy of BBR is relatively low. To overcome these drawbacks, BBR loading in NPs has been described [24].

Nanoparticles (NPs) based on synthetic biodegradable polymers, such as the FDA-approved poly(lactic acid-co-glycolic acid) (PLGA), have been widely proposed for drug delivery coupled with adjuvant therapy due to their good safety profile and efficient delivery of their molecular cargo to tumor cells [25]. Several attempts were made to formulate PGLA-based NPs to effectively deliver drugs to GBM cells, including methotrexate and paclitaxel [26], doxorubicin [27], temozolomide and DNA repair inhibitors [28], metformin/irinotecan [29], and cisplatin [30]. More advanced contributions have reported the development of specifically designed intranasal or intracranial NPs for GBM treatments [31,32]. On the other hand, only a few studies have been reported on NP encapsulation of BBR in cancer [33,34], and in particular, no published contribution has reported the development of PLGA-based NP systems to deliver BBR to glial tumor cells, even if other BBR-NP encapsulation strategies have been proposed for this purpose [24,35,36,37]. Among nutraceutical-based compounds, to the best our knowledge, only curcumin was assayed in glioma cells as actively encapsulated in PLGA-based NPs [38,39].

The water solubility of BBR hydrochloride (BBR-HCl), the most commonly used salt of BBR, is reported as relatively low (1.96 ± 0.11 mg/mL), despite the logP value of −1.5 indicating its hydrophilic nature [40]. These properties account for its low bioavailability and at the same time represent a challenge for the BBR-HCl encapsulation in NPs based on hydrophobic biodegradable polymers such as PLGA. The literature reports loading of BBR in PLGA NPs, especially by the double emulsion method [34]. Some hydrophobic salts of BBR have been described in the literature [41,42,43]. In particular, Soulie et al. studied nine BBR salts for their crystallographic status, apparent logP, and fluorescence behavior. Among these, two salts with different logP values according to Soulie et al. were chosen in the present work, BBR laurate (BBR-L) (logP −0.52) and BBR dodecyl sulfate (BBR-S) (logP 1.73), both characterized by relatively high photoluminescence quantum yields [41].

In the present study, the two selected salts were preliminarily characterized by thermal analysis in comparison with BBR HCl and encapsulated in PLGA NPs stabilized with chitosan oleate, as previously described [44]. In chitosan oleate, the polysaccharide chain is hydrophobically modified by ionic interaction with the fatty acid, resulting in an amphiphilic polymer. During the preparation of NPs, the amphiphilic polymer surrounds the solvent droplets containing PLGA, arranging on their surface with the hydrophobic moieties towards the oil phase and the chitosan chains towards the aqueous phase, allowing one to obtain, in one step, chitosan-coated NPs. NPs with a chitosan shell present the advantage of a positive charge that improves the interaction with cell membranes, triggering endocytosis [45]. The positive charge of the NPs also allows further surface modification, by easy ionic interaction, with anionic molecules able to modulate their behavior. Previous work was performed studying surface interaction with a well-known PDT dye, indocyanine green (ICG) [46]. In the present case, ionic interaction was studied for NP coating with folic acid (F). This is among the most studied ligands used to decorate NPs for active cancer targeting, for its binding with F receptor overexpressed on the surface of cancer cells [47]. This strategy has been studied for targeting many different cancer types, excluding, until recently, glioblastoma [48,49,50]. Quite recently, the conjugation of a PDT photosensitizer with F moiety has also been proposed for tumor-specific targeting [51].

In this contribution, different PLGA/chitosan-based nanoparticle formulations, with and without F decoration, were developed and tested to deliver BBR-L and BBR-S into GBM-established cells.

## 2. Materials and Methods

### 2.1. Preparation of Berberine Salts and Water Solubility Evaluation

BBR salts were obtained according to the methods described by Soulié et al. [41]. Briefly, for BBR dodecyl sulfate salt (BBR-S) preparation, a water solution of sodium dodecyl sulfate (SDS) (Sigma Aldrich, Milan, Italy) was added in a stoichiometric ratio of 1:1 to a water solution of BBR-HCl. The blend was performed while maintaining the solution at 70 °C for 20 min. The mixture was cooled to 25 °C, then placed in an ice bath to favor the formation of BBR-S crystals, then collected by vacuum filtration using cellulose acetate filters (mesh 1.2 µm, Whatman GmbH, GE Healthcare, Milan, Italy), then dried at 60 °C for 4 h.

BBR laurate salt (BBR-L) was obtained starting from a preparation of sodium laurate salt (NaLAU). Briefly, lauric acid (Sigma Aldrich, Milan, Italy) and NaOH were mixed (stoichiometric ratio 1:1) to obtain NaLAU. Then, BBR-HCl and NaLAU aqueous solutions were mixed in a stoichiometric ratio of 1:1 and maintained at 70 °C under stirring to induce the BBR-L salt formation. The obtained solution was cooled to 25 °C, and the crystals grown were collected by vacuum filtration using cellulose acetate filters (mesh 1.2 µm, Whatman GmbH, GE Healthcare, Milan, Italy) and then dried at 60 °C for 4 h [41].

The water solubility of commercial BBR-HCl and the two salts isolated, BBR-S and BBR-L, was determined as reported below.

First, 23 mg of BBR-HCl was added to 10 mL of bi-distilled water under magnetic stirring for 24 h at room temperature. After this time, the solution was centrifuged at 3000× *g* for 25 min in order to guarantee the complete separation of insolubilized BBR-HCl. The supernatant was freeze-dried, and the obtained powder was resuspended in 3 mL of acetonitrile. The final solution was analyzed by spectrophotometric analysis (UV-Vis Lamba 25, Perkin Elmer, Milan, Italy) at 425 nm to determine the BBR-HCl concentration versus a calibration curve.

The same procedure was adopted to determine the water solubility of BBR-S and BBR-L. Briefly, BBR-S (4 mg) or BBR-L (2 mg) was added to 10 mL of bi-distilled water and magnetically stirred for 24 h at room temperature. After this time, the solutions were centrifuged at 3000× *g* for 10 min in order to guarantee the complete separation of insolubilized salts. The supernatant of each salt was freeze-dried, and the obtained powder was resuspended in 3 mL of acetonitrile. The final solution was analyzed by spectrophotometric analysis (UV-Vis Lamba 25, Perkin Elmer, Milan, Italy) at 425 nm to determine the salt concentrations versus calibration curves.

### 2.2. Physico-Chemical Characterization of Berberine Salts

For the thermal characterization of BBR salts, temperature and enthalpy values were measured at least in triplicates using a Mettler STAR^e^ system (Mettler Toledo, Milan, Italy) equipped with a DSC821^e^ Module and an Intracooler device for sub-ambient temperature analysis (Julabo FT 900, Seelbach, Germany). The samples (2–4 mg) were weighed on a Mettler M3 Microbalance, placed in sealed Al pans with pierced lids, and analyzed in the temperature range 30–300 °C (heating rate β = 10 K min^−1^, N_2_ atmosphere with a flow rate of 50 mL min^−1^). The instrument was previously calibrated with indium as the standard reference.

A Mettler STAR^e^ TGA system with simultaneous DSC (TGA/DSC1) (Mettler Toledo) was used to measure the mass losses in the same temperature range used in DSC. The analyses were carried out on 3–4 mg samples in alumina crucibles with lids (N_2_ atmosphere, flow rate 50 mL min^−1^). The instrument was previously calibrated with indium as a standard reference, and the measurements were performed at least in triplicate.

Microscopic observation of samples and hot stage microscopy (HSM) were performed on salts crystals immersed in silicone oil with a Reichert (Reichert Instruments GmbH, Arnsberg, Germany) polarized light microscope equipped with a Mettler FP82HT/FP80 system (Mettler Toledo) in the temperature range 30–300 °C and with a heating rate of 10 K min^−1^. During heating, micrographs were recorded at various time intervals with a MOTICAM 2000 video camera (Motic, Milan, Italy).

A Spectrum One FT-IR spectrophotometer (64 scans with a resolution of 4 cm^−1^) (Perkin Elmer) equipped with a MIRacle^TM^ ATR device (Pike Technologies, Madison, WI, USA) was used for Fourier transform infrared (FT-IR) mid-IR spectroscopy (650–4000 cm^−1^). The crystals as such were pressed on an ATR crystal of ZnSe for the acquisition of the spectrum in transmittance mode; the spectra were collected at least in triplicate.

### 2.3. Nanoparticles Preparation

For this study, we prepared polymeric NPs characterized by a core containing the BBR salt (BBR-S or BBR-L) dispersed in PLGA and a shell of chitosan oleate.

Chitosan oleate was prepared as previously reported by Perteghella et al. [52]. Briefly, oleic acid (Sigma Aldric) ethanolic solution, at a concentration of 1% *w/v*, was added to an aqueous solution of chitosan HCl (0.1% *w*/*v*; Sigma Aldrich). Ethanol was then evaporated by nitrogen evaporation for 1 h, and by the addition of bi-distilled water, we returned to the starting volume.

NP formation was induced by emulsification and solvent evaporation using an Ultra-Turrax T18 (Janke&Kunkel, IKA Labortechnik, Staufen, Germany) at 20,400 rpm for 10 min, progressively adding a solution of PLGA (200 µL at a concentration of 30 mg/mL) and 250 µL of BBR-S (34.2 mg/mL) or BBR-L (28.9 mg/mL) in chloroform to 5 mL of chitosan oleate (2.4 mg/mL in water). An additional 5 mL of bi-distilled water was added, and chloroform evaporation was performed at room temperature under stirring overnight. The obtained NPs, loaded with BBR-S and BBR-L, were named NP BBR S and NP BBR L, respectively.

Both NP formulations were functionalized by ionic interaction of their positive charge, related to the chitosan content, with folic acid (F), by adding drop by drop under stirring a 0.2 mg/mL solution of folic acid in PBS to equal volumes of NPs diluted 5 times with bi-distilled water. The functionalized NPs were named NP BBR SF and NP BBR LF for the systems loaded with BBR-S and BBR-L, respectively.

### 2.4. Drug Loading and Encapsulation Efficacy Determination

The BBR-S and BBR-L content in NPs was determined by spectrophotometric analysis (Perkin Elmer Instrument Lambda 25 UV/Vis Spectrometer) at 425 nm. For each formulation, NP suspensions were centrifuged (3000× *g*, 10 min) to precipitate the unreacted drugs while the NPs remained in the supernatants. The obtained pellet was freeze-dried and resuspended with acetonitrile (3 mL) and spectrophotometrically analyzed. BBR-S and BBR-L content was calculated by difference with respect to the unreacted drug amounts, determined from the calibration curves.

Drug loading (*DL*%) was calculated as follows:

$$DL\;\%=\frac{encapsulated\;drug\;\left(BBR\;DS\;or\;BBR\;LAU\right)}{encapsulated\;drug+excipients\;}\times 100$$

The encapsulation efficiency (*EE*%) was calculated as follows:

$$EE\;\%=\frac{encapsulated\;drug\;\left(BBR\;DS\;or\;BBR\;LAU\right)}{drug\;used\;for\;nanoparticle\;preparation\;}\times 100$$

### 2.5. NP Characterization: Size Distribution, Concentration and Zeta Potential, DSC, FT-IR, and TEM Analysis

The qNano Gold instrument (Izon Science, Christchurch, New Zeeland) was employed to measure the size distribution and concentration of the isolated NPs using the tunable resistive pulse sensing (TRPS) principle as already reported [53,54]. Briefly, 35 μL of purified particles were analyzed with a qNano Gold instrument using a NP200 Nanopore (Izon Science) and applying 49 mm stretch, 0.1 V, and 20 mBar parametric conditions. The calibration particles (CPC100, Izon Science) were assayed before the experimental samples under identical conditions. Size and concentrations (2000 events each) were finally determined using the qNano software provided by Izon Science (Izon Control Suite version 3.1).

Zeta potential was evaluated by a Litesizer 500 Particle Analyzer (Anton Paar, Turin, Italy) in aqueous suspension.

DSC and FT-IR analyses on BBR-NPs were performed with the same experimental conditions used to characterize the BBR salts (see Section 2.2).

TEM ultrastructural analysis of NPs was carried out using a Zeiss EM900 electron microscope (Zeiss) operating at 80 kV.

### 2.6. Cell Culture and Chemicals

High-grade astrocytoma established cell line (i.e., T98G) was obtained from the American Type Culture Collection (Manassas, VA, USA); rat normal astrocytes were provided by Prof. S. Schinelli (University of Pavia, Italy), as described in [55]. Cells were routinely grown as monolayers at 37 °C in Dulbecco’s modified Eagle’s medium (DMEM) supplemented with 10% fetal bovine serum (FBS) and 100 U/mL of penicillin and 100 μg/mL of penicillin-streptomycin (all reagents from Euroclone, Milan, Italy), under atmosphere controlled at 5% CO_2_.

To stain mitochondria in living cells, MitoTracker Deep Red FM (Thermofisher, Waltham, MA, USA), a far-red fluorescent dye (abs/em 644/665 nm) was employed as follows: cells were incubated with 10 nM dye at 37 °C for 45 min and then visualized by inverted fluorescent microscope (Nikon Eclipse TS100, Tokyo, Japan) or trypsinized for cytofluorimetric analysis.

For the evaluation of the degree of membrane polarizations, JC-1 dye (Thermofisher, Waltham, MA, USA) was employed. Specifically, T98G cells, seeded at 70–80% confluence, were subjected to S NPs incubation (i.e., 3000 cells/96 well incubated with 10^7^ blank (B) or S NPs for additional 24 h); before fluorescence microscope examinations, JC-1 (1 μM) was added for 15 min at 37 °C.

### 2.7. Cell Viability Assays

Cells were seeded at a density of 10^3^ cells/well in 96-well plates in a volume of 200 μL for 24 h and then treated with different concentrations of NPs. After 24 h p.t., 20 μL of Cell Titer One Aqueous Solution (Promega, Madison, WI, USA) was added in each well and incubated for 2 h at 37 °C. Then, absorbance was measured using a microplate reader (Sunrise, Tecan, Männedorf, Switzerland) at a wavelength of 492 nm. All experiments were performed in triplicates with independent assays.

### 2.8. BBR Photo-Stimulation

Four treatment groups were considered: group 1 (control, untreated), group 2 (LED-irradiated control), group 3 (NPs-only incubation), group 4 (NPs + LED, incubated for 4 h with BBR and irradiated). After BBR incubation, cells were washed with PBS, and 1 mL of fresh culture medium was then added to each plate. A blue LED source (Safe Imager 2.0 Blue-Light Transilluminator, Invitrogen, Carlsbad, CA, USA), operating at 447 nm and 1.2 mW/cm^2^ of intensity, set for 4 min of application, was used for irradiation as previously reported [17]. All experiments were performed in triplicate independent assays.

### 2.9. Muse Cytofluorimetric Assays

A Muse Guava Cell Analyzer (Luminex, Austin, TX, USA) was adopted for analysis of Annexin V (Annexin V and Cell Dead kit) in T98G cells as untreated (NT), exposed to LED-only stimulation (LED), NPs-only administration, and with the combined LED scheme, all evaluated after 24 h p.t. For cytofluorimetric evaluations, trypsinized cells were analyzed according to the manufacturer’s specifications for each assay. Experiments were performed in duplicates or triplicates, evaluating 2000 cells for each assay.

### 2.10. Imagestream Analysis

An ImageStream MarkII flow cytometer (Amnis, Luminex) equipped with 3 lasers (100 mW 488 nm, 150 mW 642 nm, 70 mW 785 nm (SSC) was used to assay BBR-NPs, their cellular internalization, BBR, and mitochondria co-localization and JC-1 fluorescence. For the analysis of BBR-NPs’ internalization, cells were incubated in the presence of 200 μg/mL of the compound for 4 h. The growth medium was then replaced with BBR-free, fresh growth medium, and after 24 h p.t., cells were trypsinized and analyzed by flow cytometry at 60× magnification (NA = 0.9; DOF = 2.5 μm, core size = 7 μm), excited at 488 nm (Ch02, 480–560 nm, Ch width, 528/65 bandpass). MitoTracker (Invitrogen) marker was used to highlight mitochondria, exciting with a 642 nm laser. Channel 6 (745–800 nm filter) was used for scatterplot (SSC) detection, and standard sheath fluid (D-PBS, Themofisher) was adopted in all measurements. Data acquisition was performed by INSPIRE software v0.3, while data analysis was performed using the Internalization IDEAS v6.2 (Amnis, Luminex) wizard tool, according to the gating specifications.

### 2.11. Microscopy Analysis

Phase contrast and fluorescence images were obtained using an inverted microscope (Nikon Eclipse TS100) using 10, 20, and 40× objectives. For fluorescence detection, the following wavelengths were employed: 488 nm for BBR and 633 nm for the mitochondria-staining dye.

### 2.12. Clonogenic Assay

To evaluate long-term viability effects, a clonogenic survival assay was performed as described [56]. Briefly, 24 h after NPs administration and/or LED stimulation, cells were trypsinized and seeded into 6-well plates (10^3^ cells per well), incubated for two weeks at 37 °C, and then fixed with ethanol. Cells were stained with 0.5% Crystal Violet (Sigma-Aldrich), and colonies that contained more than 50 cells were automatically counted using ImageJ colony-counter (https://imagej.nih.gov/ij/plugins/colony-counter.htmL, accessed on 13 December 2022). The number of clones/well was calculated and normalized to the corresponding control samples. Each experiment was performed in triplicate.

### 2.13. Statistical Analysis

The data were analyzed using the statistics functions of the MedCalc statistical software version 18.11.6. (http://www.medcalc.org, accessed on 13 December 2022). Either Student’s *t*-test or ANOVA test was performed. Differences were considered statistically significant when *p* < 0.05.

## 3. Results

### 3.1. Characterization of the BBR Salts

The two salts were obtained with a yield of about 70% and 27% for BBR dodecyl sulfate (BBR-S) and BBR laurate (BBR-L), respectively.

The experimental water solubility of 2.03 mg/mL was in accordance with the value reported in the literature for BBR-HCl. For the other two salts, the values determined were 0.32 ± 0.02 mg/mL and 0.03 ± 0.01 mg/mL for BBR-L and BBR-S, respectively, according to the literature logP values ranking [41], which suggested a higher hydrophobic character for BBR-DS.

For physicochemical characterization of the two hydrophobic salts, FT-IR and thermal analysis were carried out.

**Figure 1:**
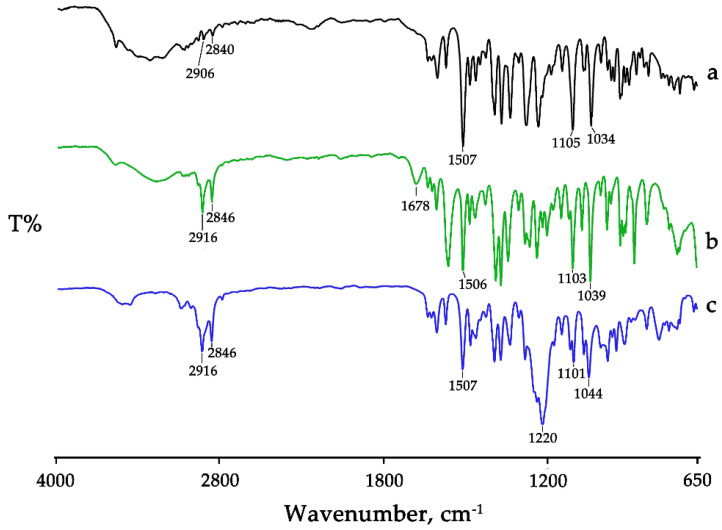
FT-IR spectra of the commercial product BBR-HCl (**a**); the salt obtained with NaLAU, BBR-L (**b**); and the salt obtained with SDS, BBR-S (**c**). Spectra are collected in transmittance reported on the Y axis versus wavenumber (in cm^−1^) reported on the X-axis.

In Figure 1, FT-IR spectra are reported in comparison with that of BBR-HCL.

In the commercial BBR-HCl (Figure 1, spectrum a), the band at 2906 and 2840 cm^−1^ was related to CH_2_ stretching vibrations, at 1507 cm^−1^ to the vibration of the aromatic C-C, at 1105 cm^−1^ to ring deformation and CH in-plane bending, and at 1034 cm^−1^ to C-H vibrations in the rings. BBR salt with NaLAU shows a similar FT-IR spectrum (Figure 1, spectrum b), with the two bands at 2916 and 2846 cm^−1^ more intensive and shifted to a higher wavenumber compared to the commercial product. These shifts together with the presence of a newly defined band at 1678 cm^−1^, due to the vibration of the asymmetric stretching of the COO- of lauric acid (shifted to lower wavenumber compared to that of the acid as such (Figure S1, spectrum a)), confirmed the isolation of BBR-L salt. Similar considerations can be extended to BBR-S salt (Figure 1, spectrum c), where the presence of the intense band around 1220 cm^−1^ due to the sulfate group recorded at a higher wavenumber with respect to the vibration of the same group in the commercial SDS (Figure S1, spectrum b) sustained the effective salification process.

**Figure 2:**
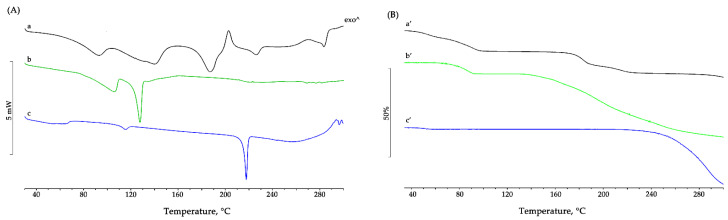
Thermal profiles recorded on the commercial product BBR-HCl (**a**,**a’**); the salt obtained with NaLAU, BBR-L (**b**,**b’**); and the salt obtained with SDS, BBR-S (**c**,**c’**). Heat flow in mW for DSC curves (**A**) and mass loss in percent for TGA curves (**B**) recorded during heating program are reported on Y-axes versus temperature in °C (X-axis).

As shown in Figure 2, the DSC curve of BBR-HCl (Figure 2A, curve a) exhibited two large endothermic effects in the temperature range between 40 and 160 °C, which peaked at 91.8 ± 0.5 °C and 140.1 ± 0.7 °C, respectively. The first one revealed the hydrating nature of BBR-HCl, as confirmed also by TG analysis (Figure 2B, curve a’), where a mass loss of 4.1 ± 0.2% between 40 and 70 °C was recorded, related to the dehydration of the active component, in agreement with the theoretical value corresponding to the loss of one molecule of water per molecule of BBR-HCl (4.8%). The second effect was related to a melting effect concomitant to a partial decomposition of the sample (loss of CO), as confirmed by the mass loss of 7.1 ± 0.2% between 65 and 100 °C recorded in the TGA curve [57].

The other endothermic peaks at 186.7 ± 0.6 °C and 283.3 ± 0.2 °C indicated that there were different melting temperatures for the crystal form of the BBR raw material. The large endothermic effect with a peak temperature of about 187 °C was accompanied by a mass loss in the TG curve (3.7 ± 0.3%), due to the dehydration of the sample losing the second water molecule present in the crystal lattice; this peak in DSC was followed by an exothermic effect that suggested a crystallization at about 202.6 ± 0.3 °C; the subsequent endothermic effect at about 225.9 ± 0.3 °C is attributable to partial decomposition (loss of CO), as confirmed by the mass loss recorded in the same temperature range in TG analysis.

The DSC curve of the BBR-L salt (Figure 2A, curve b) showed a first endothermic effect with a peak around 105 °C due to the dehydration of the sample, as confirmed by the TG curve (Figure 2B, curve b’), which in the temperature range between 60 and 100 °C showed a mass loss of approximately 6%. The next endothermic effect recorded at 127.1 ± 0.5 °C (ΔH_melting_ = 73 ± 4 Jg^−1^) was attributable to the melting of the anhydrous compound, which subsequently decomposes, as evidenced by the mass loss recorded in TG starting from 130 °C.

BBR-S preparation showed in the DSC profile (Figure 2A, curve c) a very large endothermic effect at about 62 °C, related to the dehydration of the sample, which corresponded in TG (Figure 2B, curve c’) to a mass loss of about 1% in the temperature range between 30 and 60 °C. The thermal profile then showed two endothermic effects, the first one very small (ΔH_melting_ = 6 ± 3 Jg^−1^) at 115.2 ± 0.3 °C, possibly attributable to a solid-solid transition, and the second one at 217.3 ± 0.2 °C with ΔH_melting_ of 45 ± 1 Jg^−1^, corresponding to the melting of the crystalline sample, followed by the decomposition of the melt, as evidenced by the significant loss of mass recorded in TG starting from about 215 °C.

**Figure 3:**
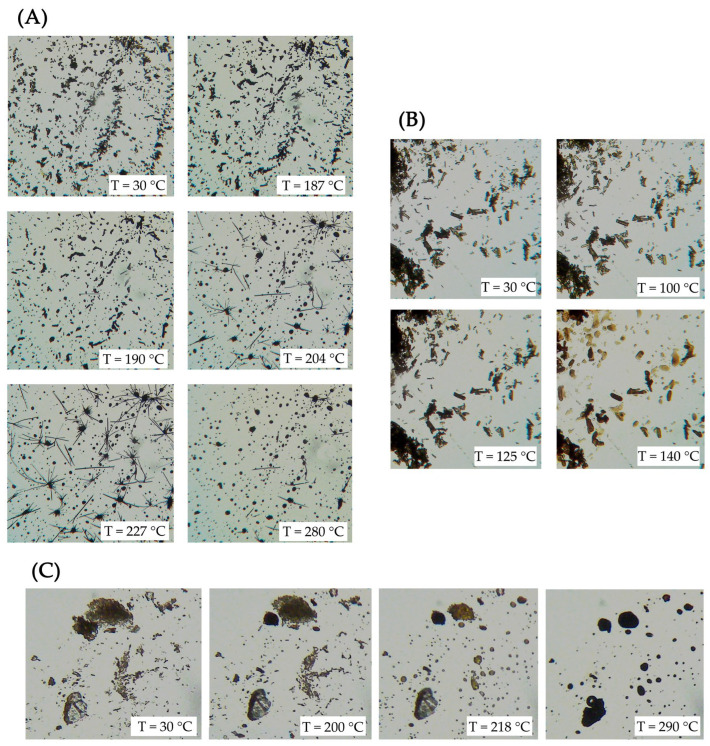
HSM photomicrographs of berberine chloride—BBR-HCl (**A**), berberine laurate—BBR-L (**B**), and berberine dodecyl sulfate BBR-S (**C**) were recorded at the various temperatures reported on the images. Photomicrographs show the morphological changes of each sample during the heating program.

Images recorded at different temperatures by the thermo-microscopic analysis were reported (Figure 3). In particular, BBR-HCl revealed the microcrystalline nature of the sample, which darkened during the heating until the melting around 187 °C. After this melting, continuing with the heating, the crystallization of a new crystalline phase occurred very clearly in needle-like crystals, which maintained their shape until melting with the decomposition of the sample at around 270 °C. For both hydrophobic salts, the microscopy analysis revealed their crystalline nature at ambient temperature, then the browning during the heating and melting at about 127 °C and 218 °C for BBR-L and BBR-S, respectively.

The thermal behavior highlighted by thermo-microscopy for the isolated salts, and especially for the commercial product, was very useful for better understanding the different thermal profiles recorded in the DSC analysis.

### 3.2. Characterization of Nanoparticles

**Figure 4:**
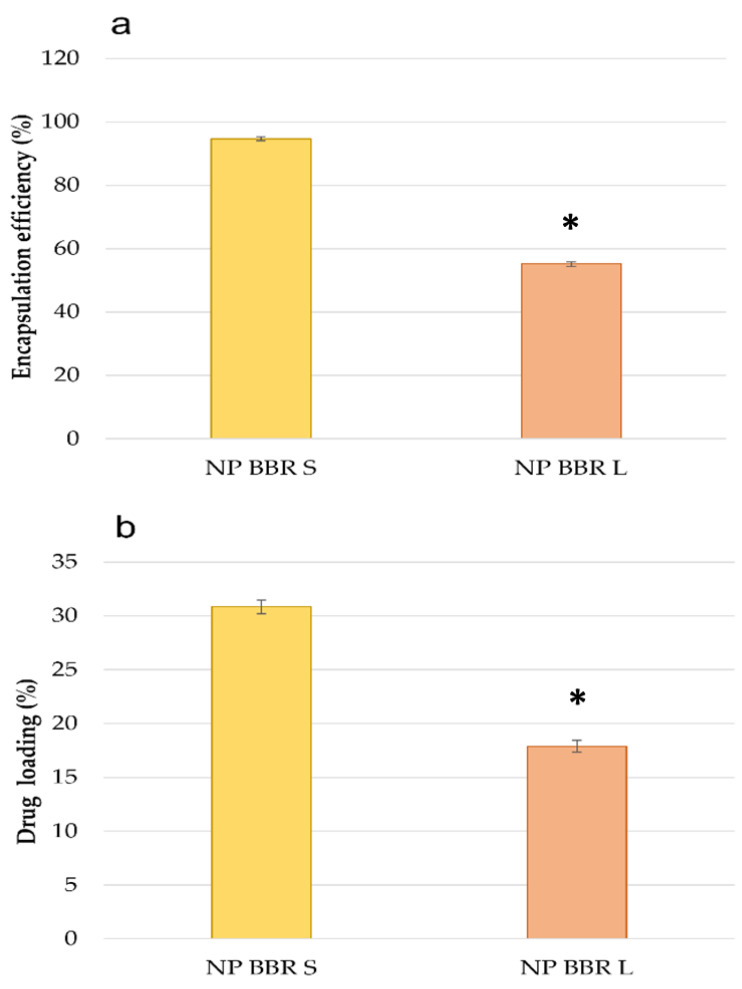
Encapsulation efficiency % (**a**) and drug loading % (**b**) for the NPs loaded with the two salts. Data are reported as mean values ± standard deviations (*n* = 3). NP BBR S vs. NP BBR L were statistically significantly different (*) for both encapsulation efficiency and drug loading (Student’s *t*-test, *p* < 0.05).

NPs were prepared with a well-known method of emulsification and solvent evaporation. As reported in the Supplementary Material, for BBR-HCl, an amount of 5 mg could not be dissolved in up to 500 µL of ethyl acetate, methylene chloride, or chloroform. The corresponding micromoles of BBR-S and BBR-L were instead easily solubilized in 250 µL of chloroform, which was conceivable considering the presence of the hydrophobic counterion moieties. Figure 4 illustrates the *EE*% (a) and *DL*% (b) values for BBR-S and BBR-L in NPs. In the literature reporting the encapsulation of BBR-HCl in PLGA NPs, the loading is generally recognized as challenging, due to the relatively hydrophilic character of the drug. Bhatnagar et al., for example, could obtain loading percentages ranging from 1.56 to 12.5% depending on the sample [34]. In the present case, the higher loading is due to the use of hydrophobic salts, having a stronger affinity than BBR-HCl for the hydrophobic PLGA. As expected, the loading of BBR-S in the hydrophobic PLGA core was higher than that of the less hydrophobic BBR-L, respecting the logP values ranking reported in the literature [41].

The use of chitosan oleate as a stabilizer of the chloroform in water nanoemulsion allowed us to obtain chitosan-coated NPs in a one-step procedure, as previously demonstrated [58].

Table 1 reports the zeta potential of the obtained NPs that, in line with the chitosan coating, had positive charge values. A clear reduction of zeta potential can be seen after the derivatization of the same NPs by the addition of a solution of the anionic folic acid. The lower zeta potential is in fact the consequence of the presence of the anionic folic acid layer around the chitosan-coated NPs. The interaction between folic acid and the cationic NPs is in fact mediated by the ionic interaction with the chitosan shell at the NPs’ surface.

**Figure 5:**
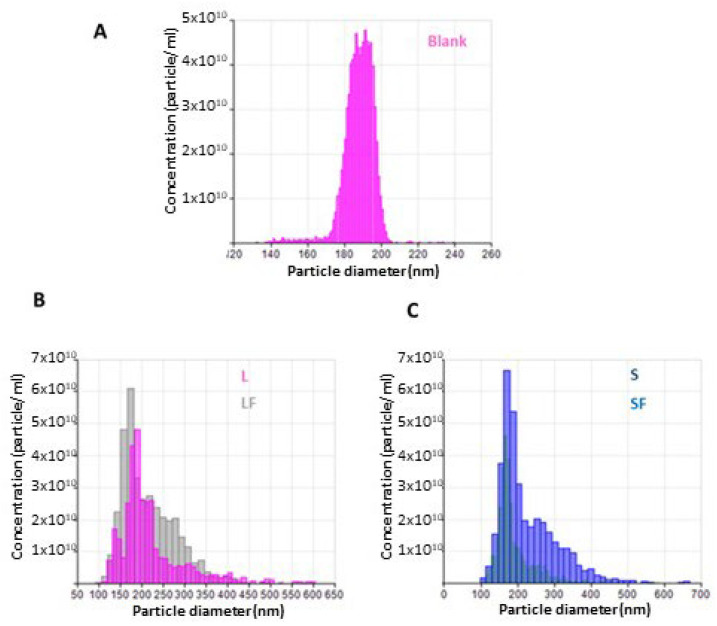
Size distribution and concentration of the investigated NPs. (**A**) Blank NPs without BBR; (**B**) NPs with BBR-L (L), NPs with BBR-L and with a folic acid coating (LF); (**C**) NPs with BBR-S (S) and with a folic acid coating (SF). Size (in nm) and concentration (particles/mL) are reported on X- and Y-axes, respectively.

To evaluate the size distribution and concentration of the investigated NPs, qNano technology (Izon) was employed. As reported in Figure 5A, NPs without any BBR cargo content (referred to as Blank) showed a unimodal size distribution with a peak average of about 190 nm, while BBR-loaded NPs (i.e., L, LF, S, and SF) displayed multimodal profiles (Figure 5B,C), and a shift of the dimensional distribution was obtained after folic acid addition, indicating the coating of folate around the NPs.

The analytical data with dimensions (nm) and concentrations (particles/mL) are summarized in Table 2.

To further characterize size distribution and to assess BBR cargo, the different NPs (i.e., L, LF, S, and SF) were visualized using Amnis Imagestream (Luminex) flow cytometry at 60× using a “high gain” modality (Figure S2). Furthermore, NPs were subjected to ultrastructural analysis by TEM, revealing, as previously highlighted by qNano analysis, different size ranges, without the presence of significant BBR crystals (Figure S3).

Physico-chemical characterization of BBR-NPs was further performed by means of DSC and FT-IR analyses to demonstrate the loading of the BBR-L and BBR-S salts on the NPs. DSC thermograms revealed the amorphous nature of the systems, confirming the homogeneous drug dispersion in the PLGA core of NPs (Figure S4).

**Figure 6:**
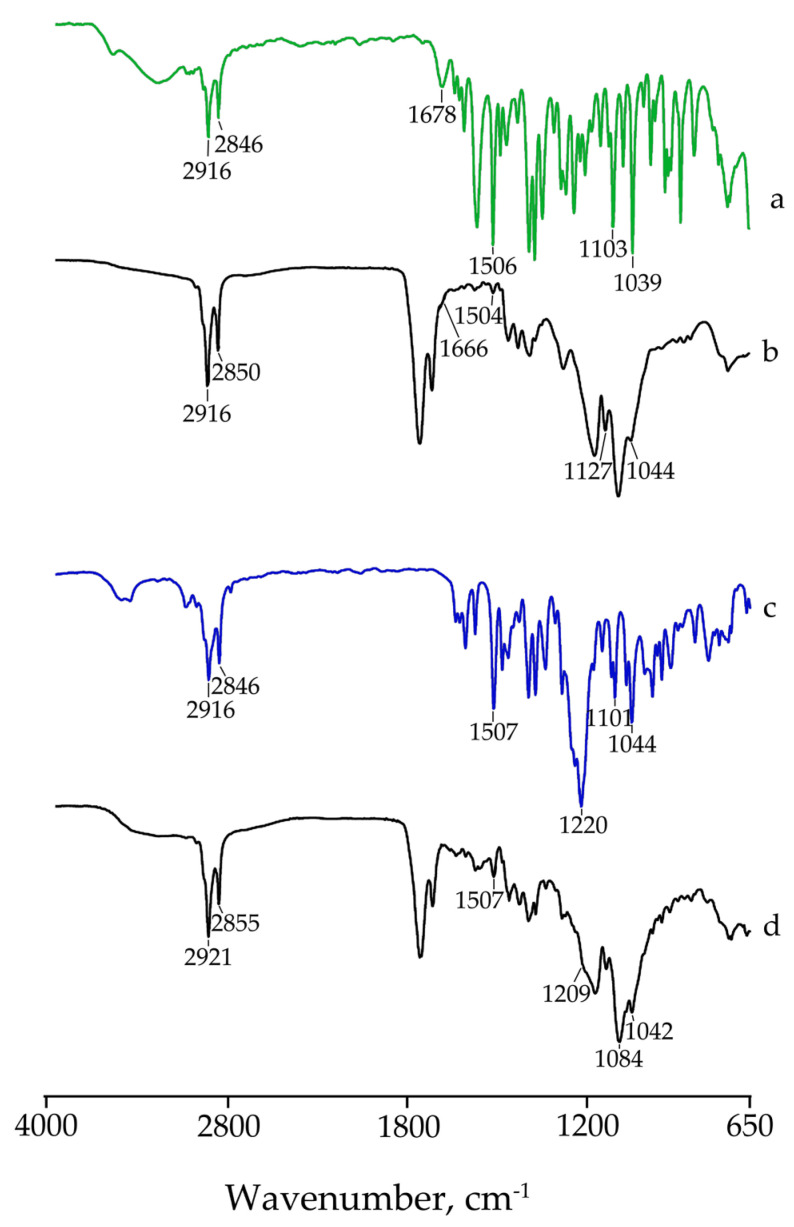
FT-IR spectra of BBR-L salt (**a**), NPs BBR-L (**b**), BBR-S salt (**c**), and NPs BBR-S (**d**).

The FT-IR analysis, which is reported in Figure 6, revealed the presence of the two salts of the drug in the BBR-NPs. In particular, some bands attributable to the vibration of characteristic groups of the BBR-L salt (e.g., the bands at 2846, 1678, 1506, 1103, and 1039 cm^−1^) shifted towards higher and/or lower wavenumbers confirmed the loading of the nano systems (Figure 6, spectrum b). Analogous considerations can be made for NPs containing BBR-S salt, in which, in this case, the characteristic bands of the salt functional groups were also recorded at higher/lower wavenumbers (Figure 6, spectrum d).

### 3.3. NP Cellular Internalization Assays

**Figure 7:**
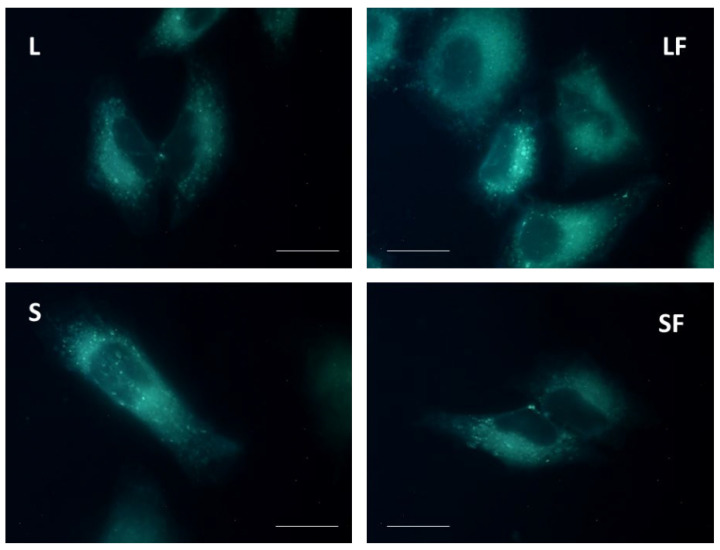
Fluorescent microscopy evaluation of BBR-loaded NPs uptake in T98G cells using Leica TCS SP8 confocal microscope at 63× magnifications (BBR exc. = 488 nm; emiss. = 500/600 nm). Scale bars = 10 μm. NPs with BBR LAU (L); NPs with BBR LAU and with folic acid coating (LF); NPs with BBR DS (S); and with folic acid coating (SF).

T98G cells were then employed to preliminarily evaluate the intracellular uptake of BBR-loaded NPs. To this purpose, 10^8^ of each NP formulation was administered to growing cells onto 24 well plates at about 70% confluence and visualized using a fluorescent inverted microscope after 24 h of incubation. As shown in Figure 7, all investigated NPs were internalized in the cytoplasm without apparent cellular cytotoxicity.

**Figure 8:**
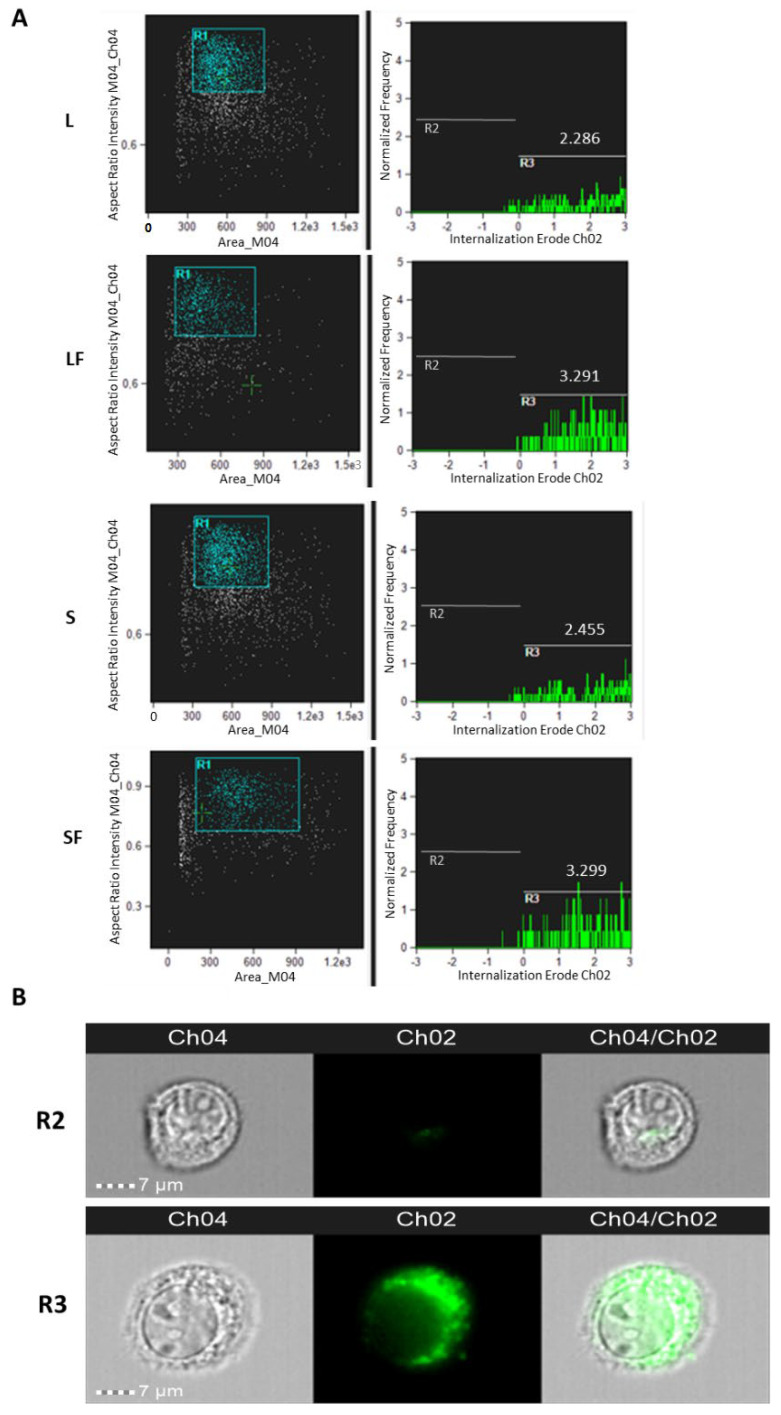
NP internalization analysis in T98G astrocytoma cells. (**A**) Amnis ImageStream flow cytometry analysis of NPs (i.e., L, LF, S, SF) internalization in T98G cells analyzed after 24 h p.t. Trypsinized cells were analyzed for the bright field (Ch04) and for BBR fluorescence (Ch02) at 60× magnification. Percentages of focused cells (*n* = 2000) with BBR internalization spots and Internalization Erode median parameters are reported. NPs’ internalization was analyzed by “Internalization” wizard with the following gating strategy: single cells were gated (using Area/Aspect Ratio Intensity, R1); Ch2 intensity was measured with “Internalization” feature and an “Erode” mask applied (gates R2 and R3). Internalization scores for R3 gated cells are reported. (**B**) Examples of cell images of R2 and R3 gates are shown.

To deeply analyze the internalization process, Amnis ImageStream (Luminex) flow cytofluorimetric technology was adopted. Specifically, 5 × 10^5^ T98G cells were placed onto 6-well plates and after 24 h of growing (reaching about 70–80% confluence) were incubated for an additional 24 h with 10^8^ of each BBR-loaded NPs. After trypsinization, BBR intracellular fluorescence was measured using 488 nm laser excitation. The fluorimetric cell gates for each assayed NP formulation and the calculated fluorescent internalization Erode profiles are reported in Figure 8, along with image galleries of R2 and R3 gated cells. The analytical results of this assay indicated that NP BBR LF and NP BBR SF displayed the highest intracellular fluorescent signals compared to the corresponding NPs without folic acid coating.

**Figure 9:**
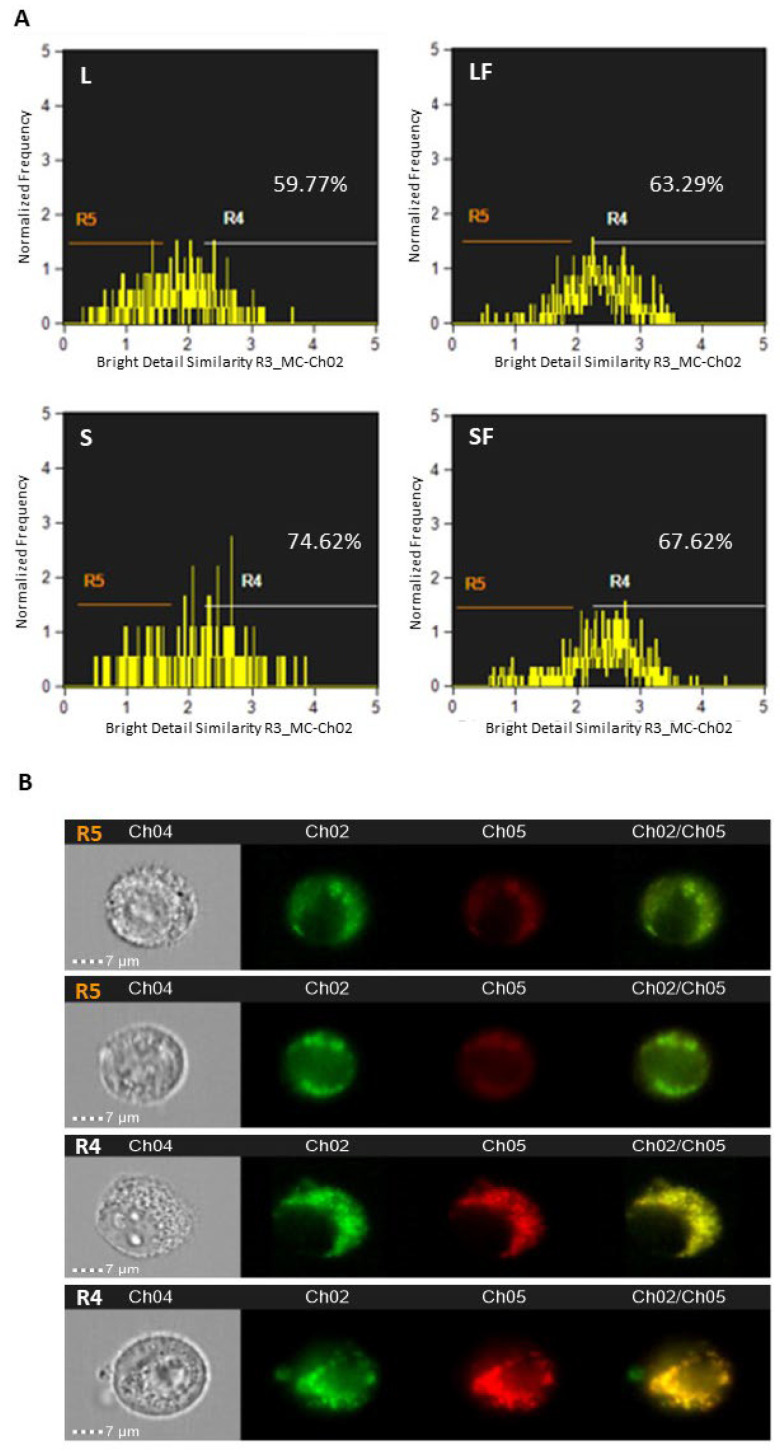
BBR-NPs and mitochondria co-localization in T98G cells. (**A**) Trypsinized cells were analyzed by Amnis ImageStream for bright field (Ch04), BBR (Ch02), and MitoTracker (Ch05) at 60× magnification. Percentages of co-localization signals (Ch02/Ch05) in focused cells at R4 gates are reported. (**B**) Examples of image cells of R5 (absence of co-localized signals) and R4 (presence of co-localized signals) gates are reported.

It was previously reported that BBR accumulated in mitochondria in astrocytoma cells [17]. In this regard, T98G cells were used to evaluate the co-localization degree within mitochondria of the different investigated BBR-loaded NPs. Thus, 5 × 10^5^ T98G cells seeded onto 6-well plates, after 24 h of incubation (reaching a 70–80% confluence) were incubated for an additional 24 h with 10^8^ of each NPs formulation (i.e., NP BBR L, LF, S, SF); before cells trypsinization, MitoTracker deep red dye (10 nM) was administered for 45 min at 37 °C. Cytofluorimetric evaluation was then performed with Amnis ImageStream using 488 and 642 lasers for BBR and MitoTracker, respectively. The results reported in Figure 9 evidenced for NPs different mitochondrial co-localization percentages, with, in particular, S and SF displaying the higher scores (i.e., 74.62 and 67.62%, respectively). On the contrary, L and LF particles showed inferior mitochondrial co-localization percentages (59.77 and 63.29%).

### 3.4. NP Cytotoxicity Evaluation

**Figure 10:**
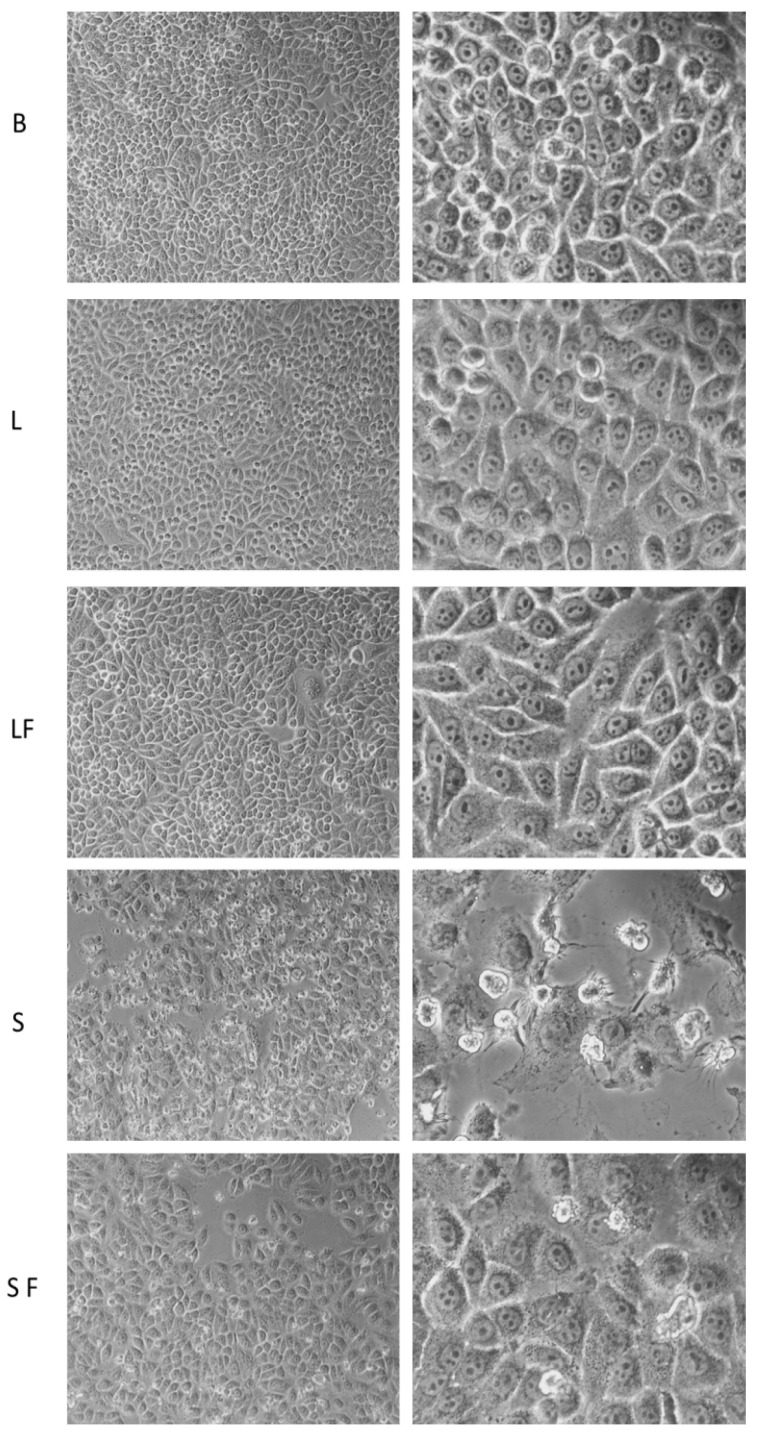
Optical microscope examination of NPs’ effect on T98G cells. NPs without BBR (B, blank), with BBR LAU (L), NPs with BBR LAU and with folic acid coating (LF), NPs with BBR DS (S), and with folic acid coating (SF) were administered (10^7^ particles) for 24 h and analyzed using a Nikon TS100 optical microscope at 10× and 40× magnifications.

T98G cells were then employed to preliminarily evaluate the cytotoxicity effect of the investigated BBR-loaded NPs. For this preliminary screening, cells were seeded at 3000 cells/96 wells and incubated for 24 h with 10^7^ particles per well following three different biological replicas. Cells were then visualized using an inverted optical microscope. As illustrated in Figure 10, NP BBR S formulation was the most effective in inducing cytotoxic effects. This result can be related to the higher hydrophobic character of this salt, responsible for its more efficient loading in the core of NPs (Figure 4).

To evaluate if long-term viability conditions confirmed the short-term results, a clonogenic evaluation of the BBR-loaded NPs on T98G cells was performed. To this aim, 10^3^ growing cells were treated with 10^7^ of each NPs formulation for 24 h, then, after media replacing, cells were cultivated for an additional 2 weeks in 6-well plates. Colonies were then stained with blue-methylene and evaluated in their numbers and distribution. As reported in Figure S5, S treatment induced a significant reduction in clones compared to other NPs, to untreated and mock (blank) treated cells (averages number of colonies with two independent experiments: NT = 842 ± 35; B = 925 ± 52; L = 783 ± 31; LF = 612 ± 42; S = 25 ± 14; SF = 799 ± 67).

**Figure 11:**
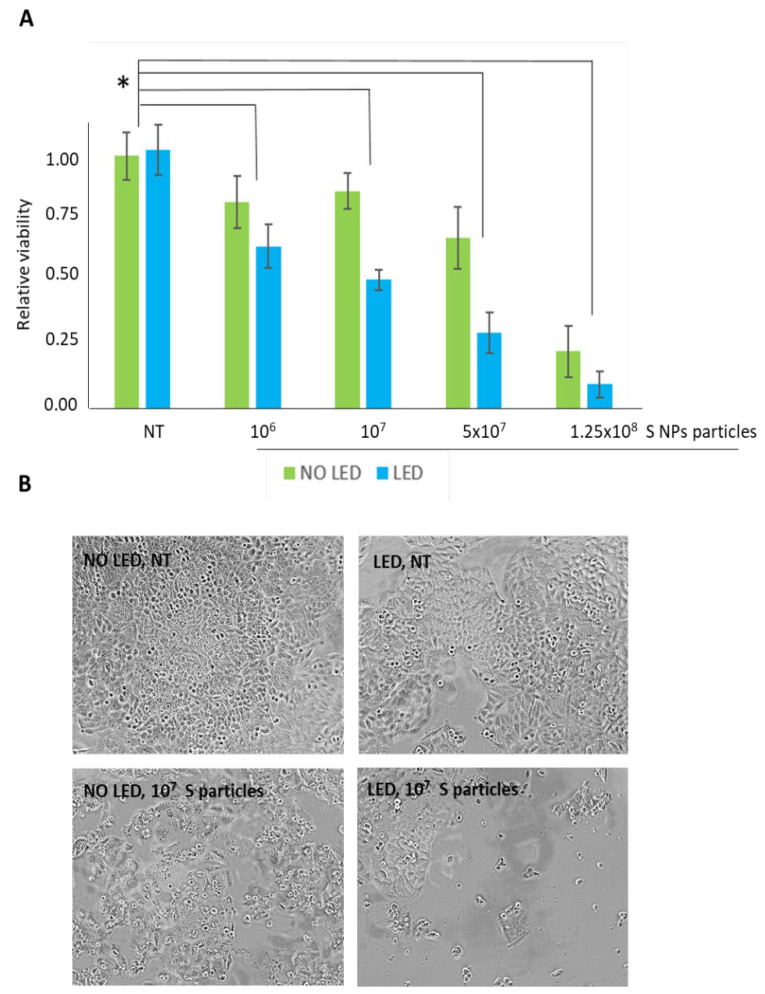
Viability effect of NP BBR S in T98G cells. (**A**) Different amounts of NP BBR S were assayed in MTS viability and compared to untreated cells (NT), in the presence or absence of LED stimulation (4 min at 447 nm and 1.2 mW/cm^2^ of intensity). The asterisk (*) indicates statistical significance compared to untreated cells (*p* < 0.05, one-way ANOVA). (**B**) Representative images comparison of untreated cells (stimulated or not with LED) and cells following 10^7^ NP BBR S administration.

Based on short- and long-term viability results, the NP BBR S formulation was selected for the next analysis, evaluating, in particular, the possibility of increasing their cytotoxic effect by photodynamic stimulation. Based on our published data [17], BBR energy stimulation was performed through a visible blue light source. Thus, T98G cells (3000 cells/96 wells in three biological replicas) were incubated for 24 h with four different concentrations of NP BBR S (i.e., 10^6^, 10^7^, 5 × 10^7^, and 1.25 × 10^8^) and exposed, or not, to light stimulation (specifically, 4 min using a blue LED transilluminator); media were then replaced and cells were further incubated for an additional 24 h at 37 °C. The above-mentioned experimental scheme was quantitatively assayed for viability using an MTS colorimetric test. As reported in Figure 11A, as previously highlighted, LED stimulation potentiated the viability reduction trend in all the NPs with a BBR S concentration administered. In addition, an NP BBR S dose-dependent effect was reported, showing that 10^7^ NP BBR S induced a roughly 50% reduction of viability in conjunction with LED stimulation. Importantly, LED stimulation in cells without NPs administration did not induce a reduction of viability. Optical microscope examinations highlighted that LED stimulation further reduced the viability of T98G cells in all assayed NP BBR S concentrations, inducing irreversible cytotoxic effects (Figure 11B).

**Figure 12:**
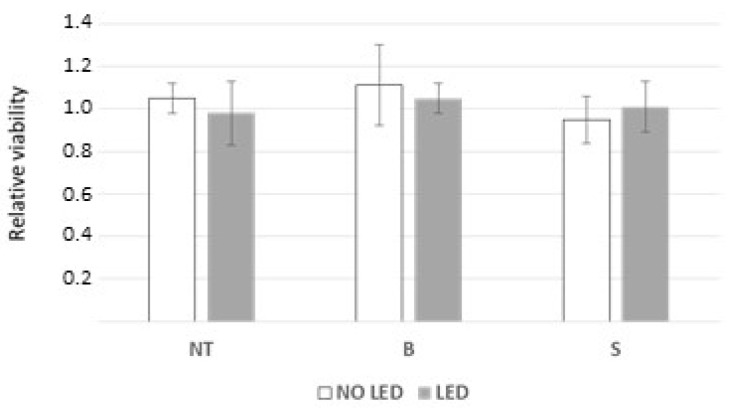
MTS viability of rat normal astrocytes: untreated (NT), after treatment with NPs BBR S (S), or with unloaded NP (B). Unloaded NPs (B) or NP BBR S (S) (both 10^7^ particles) were administered to normal rat astrocytes and analyzed by MTS assay after 24 h. Samples (in three independent replicas) were subjected or not to 4 min of light stimulation at 447 nm and 1.2 mW/cm^2^ of intensity. Data are reported as mean values of relative viability and standard deviations.

To evaluate the effect of NPs BBR S and light stimulation in non-tumor cells, normal rat astrocytes were assayed using the above-reported short-term viability protocol (i.e., 3000 cells/96 wells incubated with 10^7^ blank or NP BBR S) for an additional 24 h and subjected, or not, to 4 min light stimulation. As a result, no differences (*p* > 0.05) in MTS viability were reported; again, LED stimulation per se did not induce cytotoxicity (Figure 12).

Optical microscope examination of the same experiment confirmed the absence of cytotoxicity of the investigated particles (as blanks of NP BBR S), even in the presence of the above-described light stimulation conditions (Figure S6).

**Figure 13:**
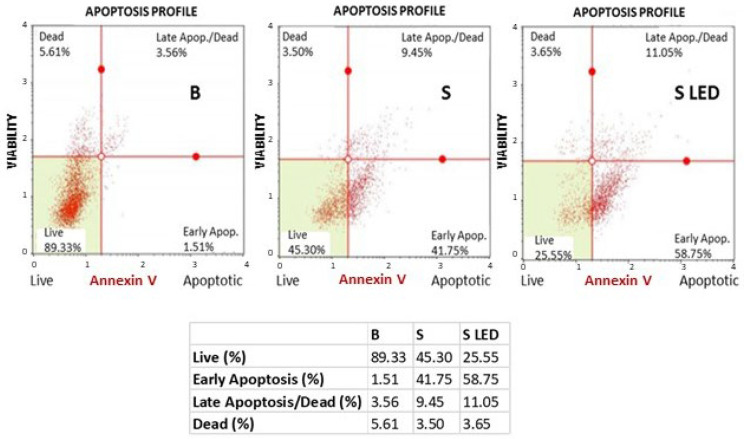
Annexin V cytofluorimetric evaluation in T98G cells. Cells (3000/96 wells) were incubated with 10^7^ unloaded NPs (B) or NP BBR S (S) for an additional 24 h and subjected or not to 4 min light stimulation (447 nm and 1.2 mW/cm^2^ of intensity). Trypsinized cells were then assayed with Muse Annexin V & Dead Cell Kit (Luminex).

Cytotoxic effects as well as molecular pathways involved in BBR and LED stimulation, following NP BBR S administration in T98G cells, were initially evaluated in terms of apoptosis activation. To this purpose, a Guava Muse cytofluorimetric Annexin V & Dead Cell Kit (Luminex) was performed on 3000 cells/96 wells incubated with 10^7^ unloaded nanoparticles (B) or NPs BBR S for additional 24 h and subjected, or not, to 4 min of light stimulation (447 nm and 1.2 mW/cm^2^ of intensity). The results, reported in Figure 13, clearly highlighted that NP BBR S induced a significant increase in early and late apoptotic events, which further increased following photoactivation. As a result, total apoptotic percentages events (i.e., early and late apoptosis) were 5.07% for mock (B), and 51.20 and 69.80% for NP BBR S and NP BBR S LED-treated cells, respectively.

**Figure 14:**
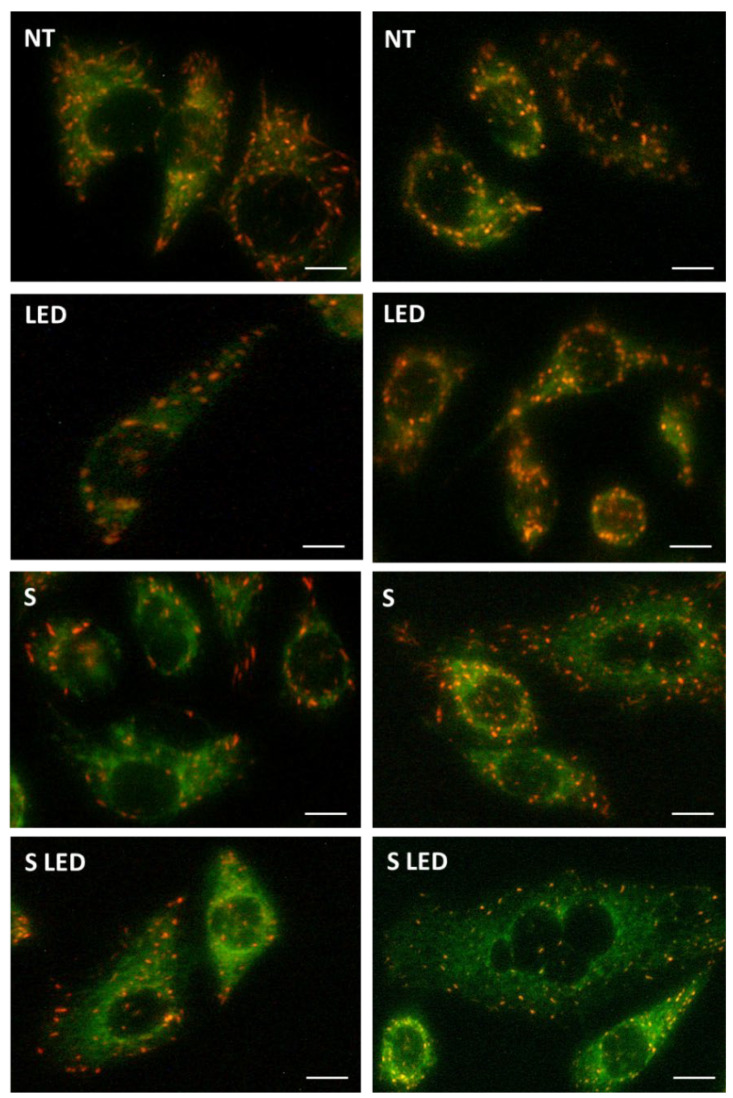
Confocal microscopy examination of JC-1 fluorescence in T98G cells. Cells (10,000/30 mm glass-dishes well) were incubated with 10^8^ unloaded NPs (B) or NP BBR S (S) for an additional 24 h and subjected or not to light stimulation (447 nm and 1.2 mW/cm^2^ of intensity); before fluorescence microscope examinations, JC-1 (1 μM) was added for 15 min at 37 °C and visualized using a Leica TCS SP8 confocal microscope at 63× magnifications (JC-1 exc. = 497/594 nm; emiss. = 527/597 nm). Scale bars = 5 μm.

Following BBR localization within mitochondria and according to the induced apoptotic activation, a physiological evaluation of the organelles (i.e., the degree of membranes’ polarizations) was assayed following NP BBR S and LED stimulation in T98G cells, through JC-1-specific cationic dye, which at low concentrations yields green fluorescence, while at higher concentrations, the dye forms aggregates that exhibit a broad excitation spectrum and an emission maximum at ∼590 nm. In detail, mitochondrial depolarization is indicated by a decrease in the red/green fluorescence intensity ratio. To this purpose, T98G cells, seeded at 70–80% confluence, were subjected to NP BBR S incubation (i.e., 3000 cells/96 wells incubated with 10^7^ unloaded NPs (B) or NP BBR S (S) for an additional 24 h); before fluorescence microscope examinations, JC-1 (1 µM) was added for 15 min at 37 °C. As reported in Figure 14, discrete red spots represented physiologically polarized organelles, while the green ones indicated depolarized mitochondria membranes, showing for this last event, in NP BBR S and NP BBR S LED treatments, a clear increase in their cellular distribution.

**Figure 15:**
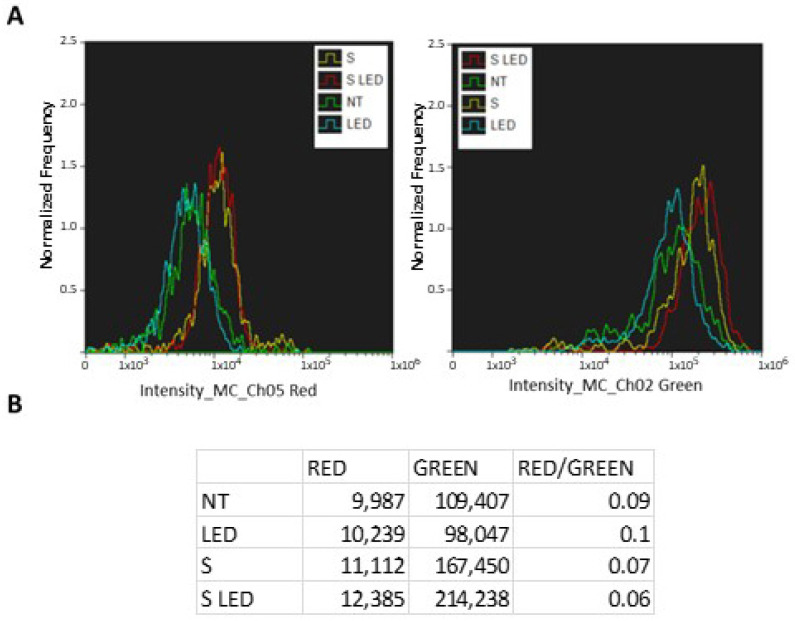
Cytofluorimetric analysis of JC-1 in T98G cells following NP BBR S administration and led stimulation. Cells (5 × 10^5^) were placed onto 6-well plates and after 24 h of growing (reaching about a 70–80% confluence) were incubated for an additional 24 h with 10^8^ of NP BBR S. (**A**) After trypsinization, JC-1 fluorescence was measured using 488 and 642 nm laser excitation in untreated (NT), LED-only treated (4 min at 447 nm and 1.2 mW/cm^2^ of intensity), NP BBR S administration (S), and combined with LED stimulation (S LED). (**B**) Fluorescence intensity (in arbitrary units) measured at Ch05 (Red) and Ch02 (Green) channels are reported with red/green ratio.

To quantify polarized/depolarized organelles, a cytofluorimetric assay was performed, assessing mitochondrial depolarization as a decrease in the red/green fluorescence intensity ratio. Cells (5 × 10^5^) were placed onto 6-well plates and after 24 h of growing (reaching about a 70–80% confluence) were incubated for an additional 24 h with 10^8^ untreated (NT) or NP BBR S and subjected, or not, to light stimulation (4 min at 447 nm and 1.2 mW/cm^2^ of intensity). After trypsinization, BBR intracellular fluorescence was measured. Amnis Imagestream technology (using 488 and 642 nm laser excitations) was used to quantify fluorescent green and red spectra, following NPs administration and LED stimulation in T98G cells. As reported in Figure 15, a significantly increased trend of depolarized mitochondria was scored following NP BBR S internalization and, mostly, after LED stimulation, compared to untreated (NT) and LED-only treated cells.

## 4. Discussion

Within brain neoplasms, glial tumors of high malignancy (i.e., GBM) are the most aggressive cancers, characterized by an extremely poor prognosis [1]. For GBM, first-line therapeutic approaches, such as surgical resection, followed by radiotherapy and chemotherapy, result nowadays in modest prognosis success [2,3]. Therefore, the search for novel experimental approaches is imperatively required [4].

Recent emerging evidence has highlighted that photodynamic therapy (PDT), a physical strategy that combines light energy stimulation with a photosensitizer (PS), is a promising anticancer approach against different tumors [59], including astrocytic cancers [60,61,62,63].

Thus, to develop effective PDT protocols, the selection of PS agents and the improvement of their cellular uptake are key milestones. Among an increasing number of natural and synthetic PSs, the plant-isolated alkaloid BBR has demonstrated a wide in vitro efficacy [64,65,66], which is also coupled with effective PDT anticancer schemes [17]. However, as we have previously reported in a large BBR kinetic study in different low- and high-grade astrocytoma cells, BBR is required to be administered at relatively high doses (above 100 μg/mL) to significantly affect cell viability [17]. Consequently, to overcome BBR’s low solubility and its poor permeability within cells, different NP formulations were designed in the present study to load BBR and improve its effectiveness.

The systems here developed are envisaged for a direct application after surgical intervention at the peritumoral area, where they can be irradiated to activate the PDT effect. This could help reduce the viability of small tumoral lesions or cells that could escape surgical removal. From this perspective, the NPs have been designed to be internalized into tumoral cells as such by endocytosis, and act after irradiation by inducing the PDT effect. This relies on ROS generation due to the release of energy by the activated photosensitizer. The formulation in NPs improves the photosensitizer penetration inside the cells and increases its concentration, especially close to mitochondria, where strong ROS generation induces cell death [67,68].

In particular, we focused on polylactide-co-glycolide (PLGA) due to its biodegradability, favorable safety profile, and approval by the Food and Drug Administration for human use [69]. For these reasons, PLGA-based NPs have been extensively employed for anticancer drug delivery approaches [70,71]. 

Chitosan oleate use in NP preparation was exploited, like in previous studies, to easily obtain chitosan-coated NPs, which can further be subjected to further ionic functionalization, in this case, performed with F.

F decoration was often proposed in the literature for the targeting of NPs to cancer cells, due to their overexpression of F receptors, and the well-known role of F to trigger receptor-mediated endocytosis [47].

In the present case, the presence of F at the NPs’ surface was obtained only by electrostatic interaction, following an approach previously proposed that involves just one easy manufacturing step [72,73]. This was made possible by the presence around the NPs’ PLGA core of a chitosan shell that was responsible for a strong positive charge, as demonstrated by the values, higher than 40 mV, of zeta potential. The addition of F decreased zeta potential, as expected by the charge neutralization, to around 10 mV. While the decorated NPs exposed F for the interaction with the cells, a shielding occurred of the chitosan coating and its strong positive charge, which has been indicated as a favorable element for internalization triggering in glioma cells as well [74,75]. In the paper of Varan and colleagues, CS-coated PCL-based NPs, despite their slightly larger dimensions, showed improved cytotoxicity of docetaxel cargo in comparison with the unloaded ones. This was in part attributed to their easier interaction with cell membranes of opposite charge. Activation of the caspase-3 mechanisms by chitosan has also been claimed, as previously highlighted [76]. Moreover, some authors have compared NPs based on pluronic F127 with and without chitosan coating and demonstrated that the chitosan utilized to decorate NPs not only could better interact with mammalian cells thanks to NPs positive charge, but it was also able to target cancer stem-like cells (CSLCs) via its specific interaction with the CD44 receptors overexpressed in these cells. This binding with CSLC was also higher than the interaction with CD44 receptors on normal human adipose-derived stem cells, envisaging a specific targeting for the CSLC cells, which are more closely involved in cancer recurrence [77].

Cell internalization assays demonstrated here that all PLGA/chitosan-based NP formulations effectively released BBR in the cytoplasm, particularly within mitochondria organelles. However, while the F coating of NPs produced better intracellular delivery against folate receptor overexpressing cancer cells [78], the most effective mitochondria-specific targeting was related to NP BBR S without folic acid coating. These NPs were also the most effective in inducing short- and long-viability reductions, probably due to the higher drug loading with respect to the BBR loaded in NP BBR L (about 17 and 30% for NP BBR L and NP BBR S, respectively). The enhanced cytotoxic effects of NP BBR S might therefore be related to their greater ability to localize within the mitochondria. This peculiar intracellular distribution of PS, in this case BBR in mitochondria, can also justify the increased cytotoxicity induced by PDT stimulation, resulting in the activation of specific cell death pathways [79,80]. Different cationic NPs have been previously developed, in which the positive charge is claimed as a key factor for the localization at the negatively charged mitochondria [68]. It was established that, in cancer cells, the inner membrane of mitochondria presents an even stronger negative potential with respect to the normal cells and can therefore electrostatically interact and aggregate with positive NPs [81].

Most of the experiments reported here used T98G cells, one of the most employed established GBM cell lines. As already shown [82], this cell line has displayed a high degree of expression of antioxidant enzymes together with low endogenous oxidative-stress levels; thus, these cells can be referred to as paradigmatic biological entities characterized by a relatively high level of intrinsic resistance to the photo-activation process. Furthermore, highly malignant derived T98G GBM cells have been employed in several in vitro PDT investigations [83,84]. While T98G cells exhibited reduced viability following S NPs administration, and particularly, following LED stimulation, normal cells (as rat primary astrocytes) did not show cytotoxicity. This different behavior might be related to a relatively inefficient cellular uptake of PS in normal rat astrocytes compared to low- and high-grade astrocytoma cells [55].

It was previously reported that BBR treatment activated apoptosis in different tumor cells [85,86,87], including GBM cell lines [17,80]. In line with this evidence, NP BBR S treatment in T98G cells induced apoptosis, as revealed by cytofluorimetric Annexin V assays. It was widely reported that the loss of plasma membrane asymmetry is an early event in apoptosis, independent of the cell type, resulting in the exposure of phosphatidylserine residues at the outer plasma membrane leaflet. To this purpose, Annexin V was shown to interact strongly and specifically with phosphatidylserine residues as an index to monitor apoptosis activation [88]. Specifically, NP BBR S administration, and in particular LED stimulation, markedly increased early apoptosis induction events in T98G cells. Based on the prominent BBR-loaded NPs’ mitochondria localization and on the activation of early apoptotic events, the membrane-permeant JC-1 dye, widely used in apoptosis studies, was adopted to monitor mitochondrial health. In fact, JC-1 dye can be used as an indicator of mitochondrial membrane potential in a variety of cell types [89]. JC-1 dye exhibits potential-dependent accumulation in mitochondria, indicated by green fluorescence emission (at ~529 nm) for the monomeric form of the probe, which shifts to red (~590 nm) with a concentration-dependent formation of red fluorescent J-aggregates. According to our results, mitochondrial depolarization was highlighted by a decrease in the red/green fluorescence intensity ratio.

## 5. Conclusions

In conclusion, despite different types of nanocarriers that have been proposed for encapsulation of BBR, those described here, particularly PLGA/chitosan NPs loaded with the hydrophobic salt berberine-dodecyl sulfate, were effective in inducing toxicity in a GBM established cell line (T98G cells) and might therefore be considered as promising examples of mitochondria-targeting nanomedicine tools, particularly suitable for the application in PDT treatments.

The encouraging results obtained here in vitro deserve to be confirmed by a direct application of the experimental protocol in in vivo models of astrocytomas. To complete the present study, therefore, in vivo experiments are scheduled along with PDT stimulations by means of specifically designed optical nanofibers. These results might represent further advances toward an effective therapeutic strategy for the treatment of malignant gliomas.

## Figures and Tables

**Table 1:** Zeta potential values of the NPs and of the same NPs functionalized with folic acid.

	Zeta Potential (mV) *		
	**Blank (Unloaded)**	**BBR-L**	**BBR-S**
without folic acid	47.6 ± 0.3	41.9 ± 3.1	43.4 ± 2.4
with folic acid	10.2 ± 0.1	6.2 ± 0.1	9.2 ± 0.2

* mean ± sd; (*n* = 3).

**Table 2:** Size and concentration data of all considered NP formulations.

NPs	Mean Diameter (nm)	Mode (nm)	Concentration (Particles × 10^10^/mL)
Blank (unloaded)	188	192	8.59
NPs BBR L	216	173	3.68
NPs BBR LF	212	189	3.39
NPs BBR S	191	167	3.76
NPs BBR SF	229	171	3.25

## Data Availability

Data are available from the corresponding author.

## Supplementary Materials

The following supporting information can be downloaded at: https://www.mdpi.com/article/10.3390/pharmaceutics15041078/s1, Table S1: Qualitative determination of BBR-HCl solubility in different organic solvents; Table S2: Qualitative determination of BBR-L and BBR-S solubility in different organic solvents; Figure S1. Spectra of sodium laurate (a) and sodium dodecyl sulfate (b). Figure S2. Flow cytometric characterization of NPS. NPs (i.e., L, LF, S, and SF) were analyzed by flow cytometry (Amnis Imagestream, Luminex). (A) High-gain-mode acquisition with 60× magnification was applied to plot particle distribution according to their size (Area_M04/Aspect Ratio Intensity), thus defining R1 and R2 gates. (B) Examples of NP image galleries within the indicated gates—channels are reported with BBR signals (BF = bright field; SC = size scatter). Figure S3. TEM ultrastructural analysis of NPs. NPs (i.e., L, LF, S, and SF) were visualized on a Zeiss EM900 electron microscope (Zeiss) operating at 80 kV. Scale bars = 100 nm. Figure S4. DSC profiles of NPs loaded with BBR-L (a) and BBR-S (b). Figure S5. Clonogenic evaluation of the BBR-loaded NPs on T98G cells. First, 103 growing cells were treated with 107 of each NP formulation for 24 h; then, after media replacing, cells were cultivated for an additional 2 weeks in 6-well plates. Colonies were then stained with blue-methylene and evaluated for their numbers and distribution. Figure S6. Optical microscope examination of NPs’ effect on rat normal astrocytes. NPs without BBR (B, blank), with BBR DS (S), with or without LED stimulation (4 min light stimulation) were administered (10^7^ particles) for 24 h and analyzed using Nikon TS100 optical microscope at 10× and 40× magnification. Figure S7. In vitro BBR-L and BBR-S release profile from nanoparticles.
