# Supplementary material for: Development of Berberine-Loaded Nanoparticles for Astrocytoma Cells Administration and Photodynamic Therapy Stimulation
Source: Pharmaceutics. 2023 Mar 27;15(4):1078. doi: 10.3390/pharmaceutics15041078 (PMC10146331; doi:10.3390/pharmaceutics15041078)
Supplement: Supplementary file 1 [file pharmaceutics-15-01078-s001.zip › pharmaceutics-2243592-supplementary.pdf]

SUPPLEMENTARY MATERIALS

Development of Berberine-Loaded Nanoparticles for Astrocytoma Cells Administration and Photodynamic Therapy Stimulation

Sergio Comincini, Federico Manai, Milena Sorrenti, Sara Perteghella, Camilla D`Amato, Dalila Miele, Laura Catenacci, and Maria Cristina Bonferoni

Berberine chloride solubility evaluation in organic solvents

Berberine chloride (BBR-HCl - Sigma Aldrich, Milan, Italy) was analyzed to evaluate its solubility in organic solvents (ethyl acetate, chloroform and dichloromethane). In particular, BBR-HCl was added to the selected solvents (concentration range 9-20 mg/mL) and maintained under magnetic stirring (400 rpm) for 4 hours at 25°C.
In detail, 5 mg of BBR-HCl were added to different volumes of ethyl acetate, chloroform and dichloromethane to determine the solubility (Table S1).

Table S1. Qualitative determination (yes/no: soluble/non-soluble) of BBR-HCl solubility in three different organic solvents. Different volumes of each organic solvent were added to 5 mg of BBR-HCl.

	Volume of organic solvent (μL)			
	250	350	450	550
ethyl acetate	no	no	no	no
chloroform	no	no	no	no
dichloromethane	no	no	no	no

Data reported in Table S1 demonstrated that BBR-HCl was not soluble in selected organic solvents at the considered concentrations.
For this study, we selected a maximum volume of organic solvents that allowed us to prepare the nanoparticles by the emulsion-evaporation method. In fact, the proposed method for nanoparticle preparation involved the dissolution of both PLGA and BBR in oil (organic) phase and the subsequent addiction of this solution to the water phase to form the final emulsion.
For this reason, we proceeded to prepare organic salts of BBR, as laurate (BBR-L) and dodecyl sulfate (BBR-S), with the aim to obtain two hydrophobic compounds which were soluble in, at least one, selected organic solvent.

Berberine salts solubility evaluation in organic solvents

The solubility of BBR-S and BBR-L salts was evaluated in ethyl acetate, chloroform, and dichloromethane. Briefly, 7.2 mg of BBR-L and 8.6 mg of BBR-S were added to 250 μL of each selected solvent at room temperature.
The salt dissolution was visually determined, and results were reported in table S2.

Table S2. Qualitative determination (yes/no: soluble/non-soluble) of BBR-L and BBR-S solubility in three different organic solvents. 250 μL of each organic solvent was added to 7.22 mg of BBR-L and to 8.6 mg of BBR-S.

	BBR-L	BBR-S
ethyl acetate	No	No
chloroform	Yes	Yes
dichloromethane	No	Yes
acetonitrile	Yes	Yes

Experimental results demonstrated that only chloroform and acetonitrile allowed us to completely solubilize BBR salts. For nanoparticle preparation, we selected chloroform because it is not miscible with water and suitable for the emulsification/evaporation method of NP preparation. Moreover, its evaporation temperature resulted in enough low to guarantee complete solvent evaporation in one night.

FT_IR characterization of lauric acid and sodium dodecyl sulfate

The FT-IT spectrum of lauric acid shows the typical bands at 2915 and 2848 cm^{-1} due to the asymmetric and symmetric CH_2 stretching vibrational frequencies, respectively. The band at 1694 cm^{-1} corresponds to the carbonyl stretching vibration of the crystalline acid, which occurs in the solid state in a dimeric form.

The same bands of the CH_2 stretching are also presented in the spectrum of the sodium dodecyl sulphate with the characteristic band at 1216 cm^{-1} due to the sulphate group (stretching vibrations of S = O bonds).

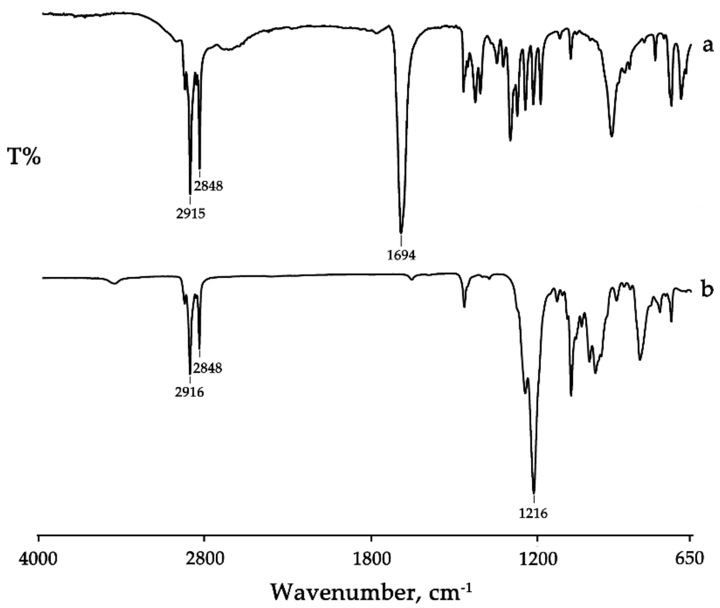

Figure S1. Spectra of sodium laurate (a) and sodium dodecyl sulfate (b).

Characterization of Nanoparticles

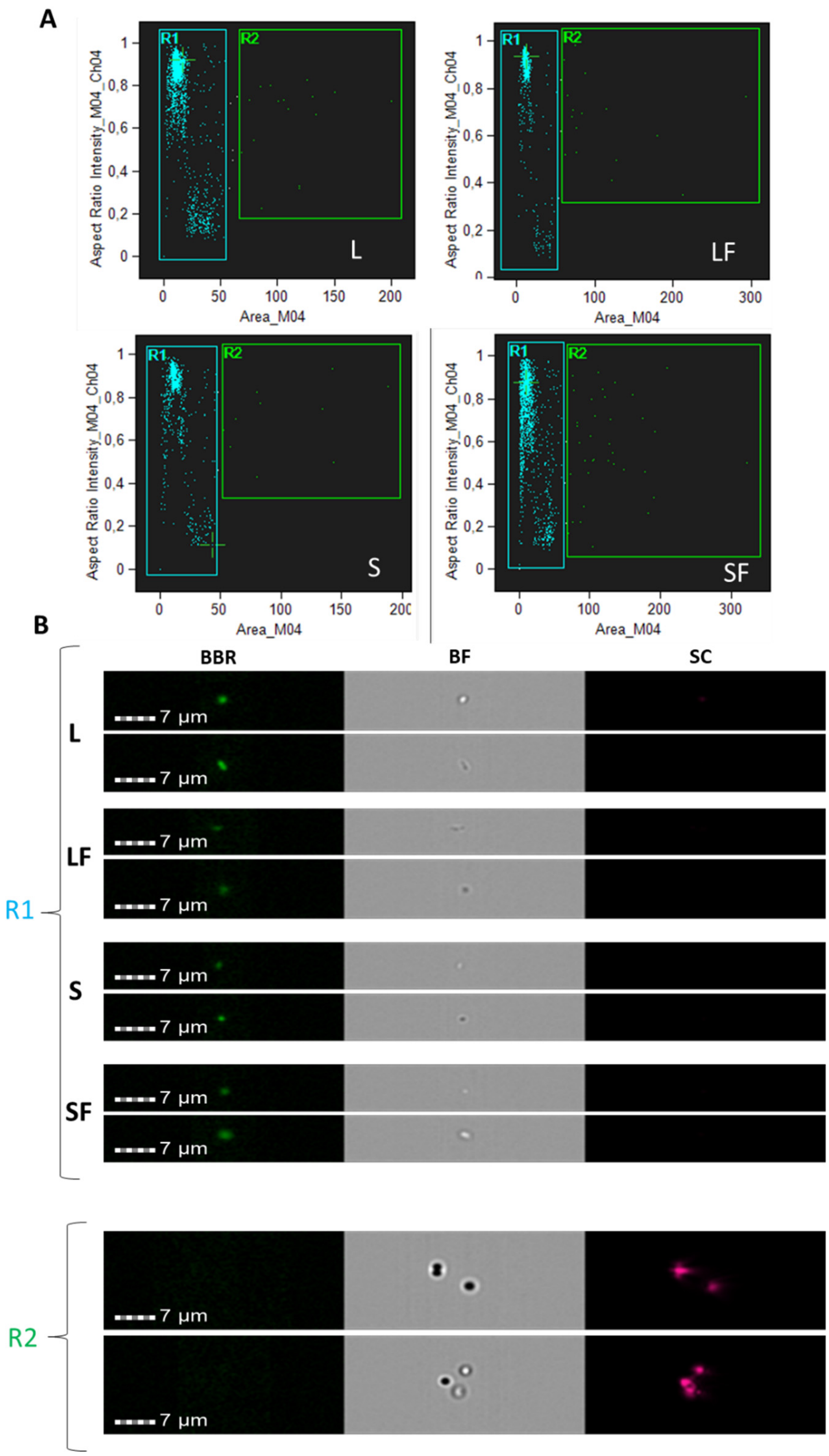

Figure S2. Flow cytometric characterization of NPS. NPs (i.e. L, LF, S and SF) were analysed by flow cytometry (Amnis Imagestream, Luminex). (A) High gain mode acquisition with 60x magnification was applied to plot particles distribution according to their size (Area_M04/Aspect Ratio Intensity) thus defining R1 and R2 gates. (B) Examples of image NPs galleries within the indicated gates-channels are reported with BBR signals (BF=bright field; SC=size scatter).

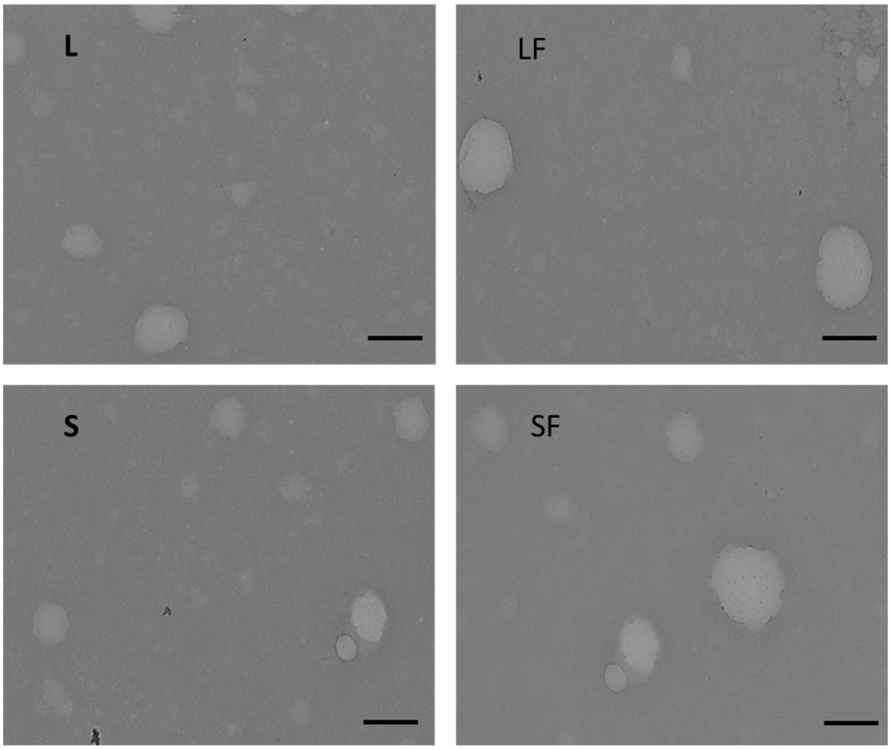

Figure S3. TEM ultrastructural analysis of NPs. NPs (i.e. L, LF, S and SF) were visualized on a Zeiss EM900 electron microscope (Zeiss) operating at 80 kV. Scale bars = 100 nm

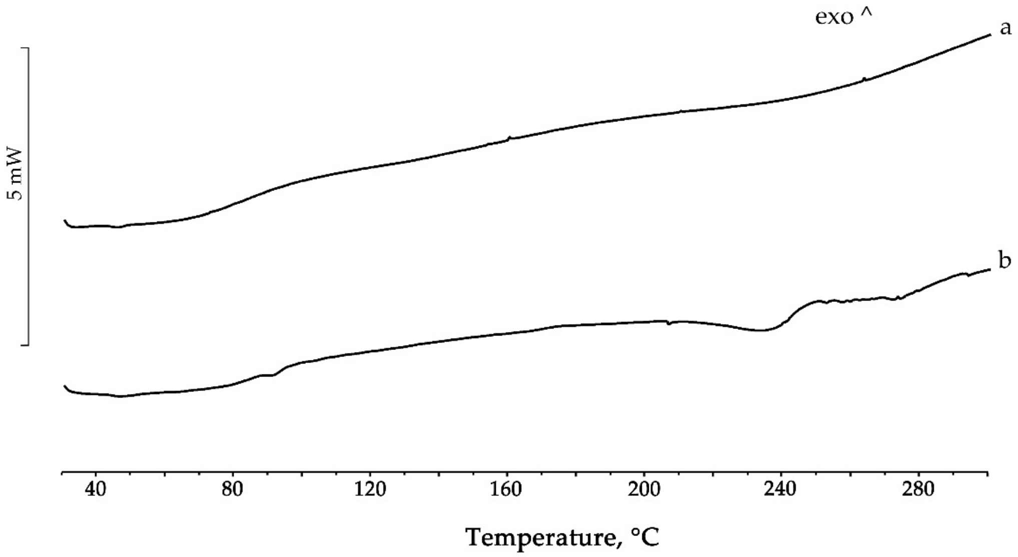

Figure S4. DSC profiles of NPs loaded with BBR-L (a) and BBR-S (b).

Clonogenic assay

To evaluate long term viability effects, a clonogenic survival assay was performed as described [41]. Briefly, 24 hours after NPs administration and/or LED stimulation, cells were trypsinized and seeded into 6-well plates (10^3 cells per well), incubated for two weeks at 37 °C and then fixed with ethanol. Cells were stained with 0.5% Crystal Violet (Sigma-Aldrich), and colonies that contained more than 50 cells were automatically counted using ImageJ colony-counter (https://imagej.nih.gov/ij/plugins/colony-counter.htmL). The number of

clones/well was calculated and normalized to the corresponding control samples. Each experiment was performed in triplicate.

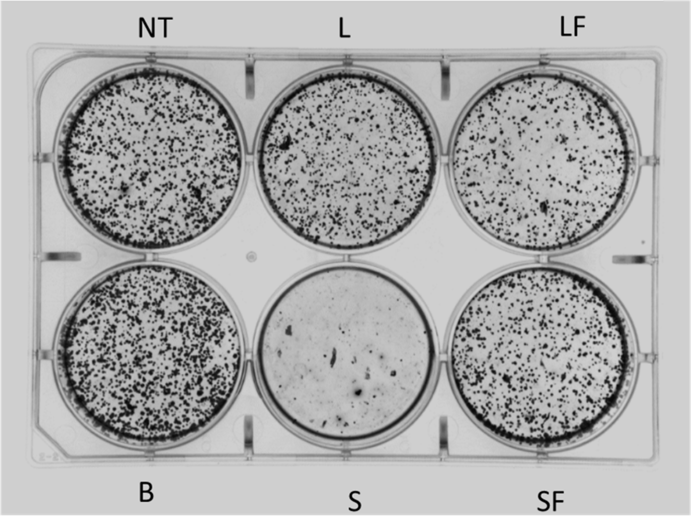

Figure S5. Clonogenic assay of NPs effect in T98G cells. Cells (103 treated with 107 of each NPs for 24 hours, after media replacing) were cultivated for 2 weeks in 6 well plates. Colonies that contained more than 50 cells were automatically counted using ImageJ colony-counter (https://imagej.nih.gov/ij/plugins/colony-counter.html).

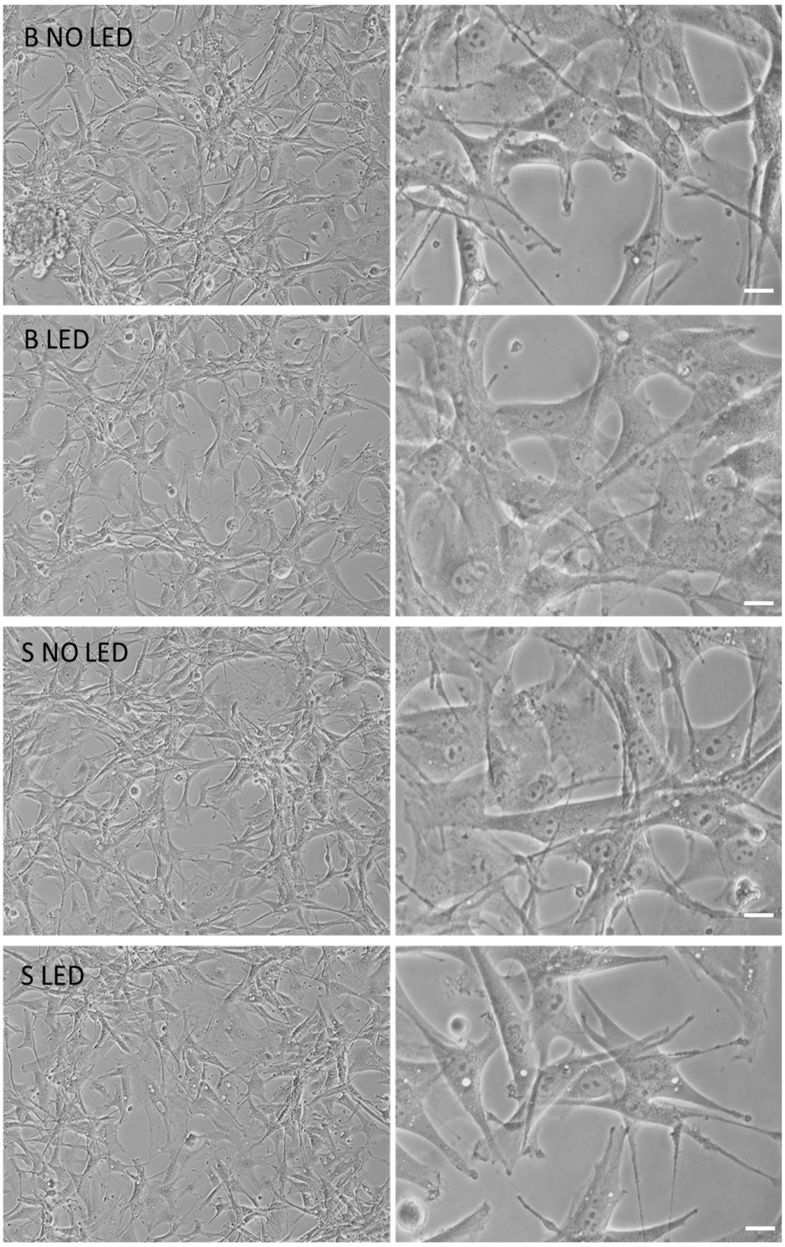

Figure S6. Optical microscope analysis of rat normal astrocytes treated with nanoparticles. Unloaded NPs (B) or NP BBR S (S) (both 107 particles) were administered to rat normal astrocytes for 24 hours and visualized using Nikon Eclipse TS100 inverted microscope at 10 or 40X magnifications, with/without led stimulation (4 minutes at 447 nm and 1.2 mW/cm2 of intensity). Scale bars=10 μm.

BBR in vitro release study

The dialysis technique was applied to investigate the BBR in vitro release as previously reported, with some modifications [J. Shaikh, D.D. Ankola, V. Beniwal, D. Singh, M.N.V.R. Kumar, Nanoparticle encapsulation improves oral bioavailability of curcumin by at least 9-fold when compared to curcumin administered with piperine as absorption enhancer, Eur. J. Pharm. *Sci. 37 (2009) 223–230.* C. Yewale, D. Baradia, S. Patil, P. Bhatt, J. Amrutiya, R. Gandhi, G. Kore, A. Misra, Docetaxel loaded immunonanoparticles delivery in EGFR overexpressed breast carcinoma cells, J. Drug Delivery Sci. Technol. *45 (2018) 334–345. B. Crivelli, E. Bari, S. Perteghella, L. Catenacci, M. Sorrenti, M.* Mocchi, S.

Faragò, G. Tripodo, A. Prina-Mello, M.L. Torre, Silk fibroin nanoparticles for celecoxib and curcumin delivery: ROS- scavening and anti-inflammatory activities in an in vitro model of osteoarthritis. Europ J Pharm Biopharm. 137 (2019) 37-45*]*. 500 µl of each nanoparticle suspension were suspended in 1.5 ml of deionized water and put into a dialysis membrane (12 kDa MWCO). Each dialysis tube was incubated in 5 ml of ethanol/water (50% v/v), maintained under mild magnetic stirring, at 37°C. At each considered time point, the BBR released was quantified by a UV-visible spectrophotometry. A calibration curve was prepared for each BBR salt (BBR-S 0.0125 – 0.2 mg/ml, r2=0.991; BBR-L 0.0325 - 0.2 mg/ml, r2=0.985). Results were expressed as mean ± standard deviation.

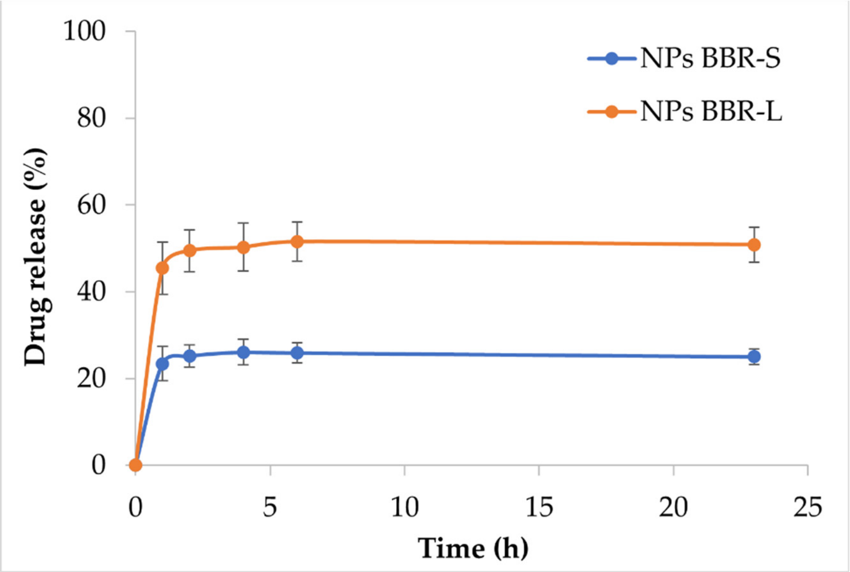

Figure S7. *In vitro* BBR-L and BBR-S release profile from nanoparticles.
